# ACAA2 Protects Against Cardiac Dysfunction and Lipid Peroxidation in Renal Insufficiency with the Treatment of S-Nitroso-L-Cysteine

**DOI:** 10.3390/biom15030364

**Published:** 2025-03-03

**Authors:** Zhengqi Xu, Feng Jiang, Xiaofan Wu, Bowen Ren, Cuntai Zhang, Li Lin, Sheng Li

**Affiliations:** 1 Division of Cardiology, Department of Internal Medicine, Tongji Hospital, Tongji Medical College, Huazhong University of Science and Technology, 1095# Jiefang Ave., Wuhan 430030, China; xuzhengqi@hust.edu.cn (Z.X.); jfdoct@hust.edu.cn (F.J.); wuxiaofan@hust.edu.cn (X.W.); renbowen@hust.edu.cn (B.R.); linli@tjh.tjmu.edu.cn (L.L.); 2 Key Laboratory of Vascular Aging, Ministry of Education, Tongji Hospital, Tongji Medical College, Huazhong University of Science and Technology, 1095# Jiefang Ave., Wuhan 430030, China; ctzhang@tjh.tjmu.edu.cn

**Keywords:** lipid peroxidation, cardiovascular disease, renal insufficiency, acetyl-CoA acyltransferase 2, S-nitroso-L-cysteine

## Abstract

The key fatty acid β-oxidation protein acetyl-CoA acyltransferase 2 (ACAA2) plays a significant role in myocardial lipid peroxidation and cardiac dysfunction induced by renal insufficiency. However, the mechanisms of lipid metabolism related to renal insufficiency-associated cardiac dysfunction remain poorly understood, and current clinical treatments have been largely ineffective. Through analysis of the Gene Expression Omnibus (GEO) database, we identified that the cardiac functional changes caused by renal insufficiency were primarily centered around the fatty acid β-oxidation signaling pathway, where ACAA2 plays a pivotal role in fatty acid β-oxidation, the tricarboxylic acid cycle, and ketone body metabolism. In an adenine-induced renal insufficiency mouse model, further examination with hematoxylin-eosin staining, Masson staining, and Oil Red O staining revealed alterations in the heart and kidney as well as the accumulation of lipid. Non-invasive blood pressure measurements and ultrasound images demonstrated improvements of peripheral vascular and right ventricular hemodynamic parameters with S-nitroso-L-cysteine (CSNO) inhalation therapy. In cell experiments, knocking down ACAA2 led to accumulation of lipid droplets and exacerbation of oxidative stress in cardiomyocytes, while overexpression of ACAA2 reversed these effects. The transcription factor FOXO4 was found to regulate lipid peroxidation by modulating ACAA2, and knocking down FOXO4 partially restored the expression of ACAA2, reducing oxidative stress in cardiomyocytes. Furthermore, exogenous CSNO effectively restored the expression of ACAA2 and reduced the level of FOXO4, thereby mitigating lipid peroxidation and improving cardiac function. Therefore, in the context of renal insufficiency, regulating the FOXO4–ACAA2 axis through CSNO inhalation therapy may provide a novel therapeutic strategy for alleviating myocardial lipid peroxidation and improving cardiac function.

## 1. Introduction

In recent years, chronic kidney disease (CKD) has increased the incidence of cardiovascular diseases (CVD) significantly. CKD induces cardiac remodeling [1], including phenomena such as myocardial hypertrophy, fibrosis, and cardiomyocyte apoptosis. The heart is a high-metabolic-rate organ that primarily relies on fatty acid oxidation to supply energy [2]. CKD and CVD share many common mechanisms of metabolic remodeling [3], including changes in fatty acid and glucose utilization as well as mitochondrial dysfunction. Studies have shown that cardiovascular events, particularly heart failure, significantly increase the risk of CKD patients progressing to kidney failure requiring replacement therapy (KFRT) [4,5]. Other studies have also revealed the important roles of gut microbiota metabolites, such as N,N,N-trimethyl-5-aminovaleric acid TMAVA [6], in myocardial hypertrophy and heart failure. The important role of uremic toxins in myocardial hypertrophy and fibrosis was also demonstrated [7,8].

Cardiac lipid peroxidation refers to myocardial dysfunction caused by the excessive accumulation of fatty acids and their metabolic products in cardiomyocytes. It plays a significant role in heart failure, particularly in heart failure with preserved ejection fraction [9,10], HFpEF, and diabetic cardiomyopathy DCM, but its impact on cardiac function in the context of uremia remains unclear. Studies have shown that adipsin improves fatty acid β-oxidation by inhibiting the mitochondrial translocation of Irak2 [11], the DPP-4 inhibitor [12] evogliptin alleviates lipotoxicity and improves mitochondrial function, Sirt5 [13] promotes fatty acid oxidation through desuccinylation, and novel ERR [14] agonists enhance cardiac metabolism, all of them significantly improving cardiac function by improving fatty acid metabolism in different models. Further research [15] indicated that cardiac-specific deletion of ACC2 increases fatty acid oxidation, maintaining cardiac health by regulating Parkin-mediated mitophagy. Additionally, some studies have explored the complex role of epicardial adipose tissue (EAT) [16] and myocardial lipotoxicity in HFpEF and obesity-related heart disease. Regulating lipid metabolism pathways and promoting the efficiency of myocardial lipid metabolism may provide emerging strategies to alleviate cardiac dysfunction in the context of chronic kidney disease.

Forkhead box O4 [17], i.e., FOXO4, is a member of the FOXO transcription factor family that regulates the expression of various genes involved in several biological processes [18], including cell cycle, apoptosis, and metabolism. In fatty acid metabolism, FOXO4 plays a role in adipose tissue by influencing the insulin signaling pathway [19]. The activation of FOXO4 can affect cellular responses to oxidative stress. For example, in the absence of insulin and IGF-1 signaling, sustained activation of FOXO4 may lead to metabolic abnormalities [19]. Additionally, FOXO4 interacts with other proteins to regulate its activity [20], modulating the cell cycle and apoptosis. Acetyl-CoA acyltransferase 2 (ACAA2) is an acyl-CoA acyltransferase that participates in the final step of fatty acid β-oxidation. In tumor cells, high expression of ACAA2 is associated with neuroendocrine phenotypes of cancer [21]. Furthermore, the role of ACAA2 in fatty acid oxidation provides protective effects in organs such as the liver and kidney. For instance [22], during acetaminophen-induced hepatotoxicity, upregulation of ACAA2 can enhance mitochondrial fatty acid oxidation, thereby reducing liver damage.

S-nitrosylation is an important post-translational modification of proteins, playing a crucial role in regulating cellular signal transduction, protein function, and cellular metabolism [23]. S-nitrosylation involves the addition of a nitric oxide NO group to cysteine residues within proteins, forming S-nitrosothiols. This modification can significantly influence protein stability, activity, and protein–protein interactions. CSNO, i.e., S-nitroso-L-cysteine, has been shown in previous studies to effectively improve cardiac function in diabetic mice following aerosol inhalation [24]. Additionally, studies have demonstrated that S-nitrosylation plays a critical role in cardioprotection [25,26], particularly in myocardial ischemia–reperfusion injury. Through modulating calcium handling in cardiomyocytes, regulating mitochondrial function, reducing reactive oxygen species ROS production, and mitigating myocardial injury, S-nitrosylation exerts protective effects on the heart. Furthermore, it has been reported that S-nitrosylation can modulate mitochondrial respiration and energy metabolism by modifying subunits of the mitochondrial respiratory chain complex. However, some studies also suggest that nitrosylation modifications in the heart may impair cardiac function, and the specific role of thiol S-nitrosylation remains unclear.

Therefore, as the metabolic mechanism of myocardial lipid peroxidation based on renal insufficiency remains unclear, and no studies have explored the specific role of CSNO in this regard, we propose that the ACAA2 protein exerts a protective role against myocardial lipid peroxidation in renal dysfunction and that CSNO participates in protecting against cardiac dysfunction under renal insufficiency through the FOXO4–ACAA2 axis.

## 2. Materials and Methods

### 2.1. Bioinformatics Analysis

To better obtain key data from the gene expression profiles, we downloaded RNA-seq data [27] (GSE106385) from CKD mice and healthy controls through the GEO database (https://ncbi.nlm.nih.gov/geo/ (accessed on 8 May 2023)) and performed a series of bioinformatics analyses, including the following: search for differential genes using the online websites (https://www.networkanalyst.ca/ (accessed on 10 May 2023)); identification of statistically significant differentially expressed genes DEGs based on differences in expression values between samples; construction of the ridgeline graph, enrich net, and volcano graph using the online tool to identify overlapping modules and genes in the dataset; construction of the protein–protein interaction PPI network related to fatty acid β-oxidation by analyzing the differentially expressed genes using the STRING database (https://cn.string-db.org/ (accessed on 5 June 2023)); visualization of the results with Cytoscape software 3.9.1; prediction of the upstream transcription factors of ACAA2 using the JASPAR database (https://jaspar.elixir.no/ (accessed on 8 December 2023)); and preliminary construction of a molecular docking model using PyMOL software 2.5.5.

### 2.2. Animals and Adenine-Induced CKD Model

A total of 30 C57BL/6 mice (18–22 g, 6–7 weeks, male) were purchased from Beijing Vital River Laboratory Animal Technology (Beijing, China). After one week of adaptation, the C57BL/6 mice were randomly divided into three groups: Control, CKD, and CKD + CSNO groups (*n* = 10). The random numbers for animal grouping were generated by the “Rand()” function in Microsoft Excel. All animal experiments were approved by the Animal Care and Use Committee of Tongji Hospital, Tongji Medical College, Huazhong University of Science and Technology (Approve number: TJH-202306049). Mice had free access to food and water and were housed in a specific pathogen-free room with a 12 h light/dark cycle at a temperature of 25 ± 1. Based on the results of the pre-experiment and the current literature report, for CKD modeling, C57BL/6 mice received an adenine gavage (50 mg/kg/d, in saline, purity ≥ 98.0%, HY-B0152, MCE) for a total of 4 weeks. The CKD + CSNO group mice received nebulized inhalation of CSNO from the week 3 to 8 (from the 15th day) (88 ppm for 20 min per day). The mice in the control group were administered the vehicle in parallel. Dynamic monitoring was performed by non-invasive cardiac ultrasound. After 6 weeks of treatment, the mice were sacrificed after intraperitoneal injection of pentobarbital, and the tissues and blood samples were collected for further data analysis. CKD and CSNO administration methods were based on existing protocols in their respective settings.

### 2.3. Cell Culture and Treatment

AC16 cells were acquired from the Cell Bank of the Chinese Academy of Sciences (Shanghai, China) and cultured in high-glucose Dulbecco’s modified Eagle’s medium (DMEM, KeyGEN BioTECH, Nanjing, China) supplemented with 10% fetal bovine serum (FBS, Gibco, Waltham, MA, USA) and 1% penicillin/streptomycin (Sangon, Shanghai, China) at 37 °C with 5% CO_2_. AC16 cells were treated with different concentration of Indoxyl Sulfate (IS, purity ≥ 98.0%, I3875, Sigma-Aldrich, Saint Louis, MO, USA) to induce in vitro cardiomyocyte injury for 24 h while starving. Meanwhile, CSNO (dissolved and mixed for 5 min until orange) was added to cells with IS treatment. The above experiments were repeated three times independently.

### 2.4. Real-Time PCR

Total RNA was extracted from tissue samples using the RNA Isolation Kit (Vazyme, Nanjing, China) or from cultured cells using TRIzol (GIBCO Life Technology, Thermo, Waltham, MA, USA). After DNase treatment, first-strand cDNA was prepared from RNA (1.5 μg) using Master Mix (Life Technologies, Waltham, MA, USA). cDNA was diluted and amplified using Powerup SYBR Green qPCR Master Mix on Real-Time PCR instrument. Primers were designed using NCBI/Primer-BLAST (Supplementary Table S1). Data were normalized to a reference gene (GAPDH) and presented as the fold increase compared with RNA isolated from the control group using the 2^−ΔΔCT^ method.

### 2.5. Western Blotting to Detect Protein Expression

Tissues and cells were homogenized with precooled RIPA lysis buffer containing 1 mM protease inhibitor and 1 mM phosphatase inhibitor for 1 h. After being centrifuged at 12,000 rpm for 20 min at 4 °C, the supernatant was collected. A BCA protein assay kit was used to measure the protein concentration. Equal amounts of protein were separated by 10–12% SDS-PAGE and transferred onto PVDF membranes. The membranes were blocked with 5% skim milk for 1 h and incubated with different primary antibodies at 4 °C overnight. The secondary antibody (1:5000) was incubated for 1 h at room temperature. An ultrahigh sensitivity ECL kit (HY–K1005; MedChemExpress, Princeton, NJ, USA) was used for protein detection, which was performed on a ChemiDoc-It 510 Imager with VisionWorks software 1.6.5 (Ultra-Violet Products Ltd., Cambridge, UK).

### 2.6. Measurement of ATP Contents

The ATP levels were measured using an enhanced ATP assay kit (S0027, Beyotime, Shanghai, China). After removing the culture medium, we added 200 µL lysis solution to each well of a 6-well plate to lyse the cells and then centrifuged at 4 °C at 12,000× *g* for 5 min. The ATP standard solution was thawed on ice and diluted with ATP assay lysis solution to create an appropriate concentration gradient (0.01, 0.03, 0.1, 0.3, 1, 3, and 10 µM). Each sample or standard required 100 µL of ATP detection working solution, which was added to a light-shielded 96-well plate after preparation. The plate was left at room temperature for 3–5 min to ensure complete consumption of baseline ATP. Then, 20 µL of either the sample or standard was added to each well, mixed immediately, and measured using the Gen5 software 3.12 on the BioTek multi-function microplate reader (BioTek Instruments, Inc., Winooski, VT, USA). The obtained RLU value was converted into the corresponding ATP (µM) value based on the standard curve. Additionally, calibration using the BCA protein assay kit (mg/mL) was performed to obtain the ATP concentration in nmol/mg protein.

### 2.7. Measurement of Cell Viability

Cell viability was assessed with the CCK-8 assay kit (RM02823, ABclonal, Wuhan, China). In brief, AC16 cells (5 × 10^3^ cells/well) were plated in medium (100 μL/well) into 96-well plates with six replicate wells. After being attached, they were modeled and administered with the corresponding drugs. Following treatment, AC16 cells were incubated with 10% CCK8 for 1 h in the dark. The absorbance at 450 nm was recorded on a microplate spectrophotometer.

### 2.8. Assessment Reactive Oxygen Species

To detect the level of intracellular reactive oxygen species (ROS), dihydroethidium (DHE. cat.no.HY-D0079, MedChemExpress, Princeton, NJ, USA), a superoxide indicator, was used. For preparing DHE staining solution, we avoided light and use it immediately after preparation. Aliquoted DHE staining reagent was thawed at room temperature and diluted with DMEM. Next, 1 mL DHE staining solution (1×) was prepared per well on a 6-well plate and thoroughly mixed. It was incubated at 37 °C away from light for 30 min and then observed under a fluorescence microscope (Bx53, Olympus, Tokyo, Japan). The culture medium was removed and the cells washed once with PBS, which was then replaced with fresh DMEM, and observation under the microscope continued. The fluorescence intensity of the same cell mass was quantified using Image J 1.54 to obtain RFU values. The procedure was repeated at least three times.

### 2.9. Mitochondrial Permeability Transition Pore (mPTP) Detection

Mitochondrial permeability transition pore (mPTP) detection was performed using an mPTP assay kit (C2009S, Beyotime, Haimen, China). The cells were seeded in culture dishes and treated according to the experimental design. The culture medium was removed and the cells washed 1–2 times with PBS. An appropriate volume of mPTP detection working solution (Calcein AM + CoCl_2_, final concentration 1×) was added and gently shaken to ensure the dye evenly covered all the cells. The mixture was incubated at 37 °C in the dark for 30–45 min. After incubation, the medium replaced with fresh pre-warmed (37 °C) culture medium, and the mixture was incubated again in the dark for another 30 min to ensure intracellular esterases fully hydrolyzed the Calcein AM to generate green fluorescent Calcein. The culture medium was then removed, the cells washed 2–3 times with PBS, and detection buffer added; green fluorescence was observed under a fluorescence microscope (Wuhan, China).

### 2.10. Immunofluorescence and BODIPY Staining

AC16 cells were seeded on coverslips. After treatments, the cells were fixed with methanol for 15 min at room temperature and subsequently permeabilized and blocked with 0.5% Triton-100 and 5% goat serum in PBS for 1 h at room temperature. Then, the cells were incubated with primary antibodies diluted overnight at 4 °C. After washing three times with PBS, the cells were incubated with fluorescence secondary antibody for 1 h. As for the double fluorescence staining of BODIPY and ACAA2, the slides were evenly covered with the green fluorescent fatty acid probe solution (C2055, Beyotime, Haimen, China) after the secondary antibody incubation and incubated at 37 °C for 15 min. Then, the cells were washed three times again, and DAPI-containing anti-fluorescence quencher was used to seal the glass slides. Finally, slides were examined using a confocal microscope (Nikon, Tokyo, Japan). The primary antibodies used in this study targeted endogenous ACAA2 (1:100, A15778, ABclonal, Wuhan, China) and FOXO4 (1:100, 3307, ABclonal, Wuhan, China).

### 2.11. Transfection

Approximately 18 h before transfection, cells were seeded. Opti-MEM serum-free culture medium was added to a sterile centrifuge tube and gently mixed with an appropriate amount of ExFect. Opti-MEM was added to another sterile centrifuge tube along with an appropriate amount of DNA. ExFect–opti-MEM was added to the DNA–opti-MEM and left sitting at room temperature for 15–20 min before transfection. The ExFect/DNA complex mixture was added into the culture medium, with gentle shaking of the medium to evenly disperse the ExFect/DNA. After overnight cultivation for 24–48 h, the cells were collected for subsequent experiments. Knockout ACAA2 or FOXO4 gene expression by siRNA (RiboBio genOFF™ siRNA, Guangzhou, China) was performed using Lipofectamine 2000. Lipo3000 was used for plasmid transfection of ACAA2 overexpression plasmids (Genomeditech, Shanghai, China). After 6 h, the culture medium was replaced and the cells treated with indoxyl sulfate for 24 h while starving. The efficiency of gene knockout was evaluated through Western blotting and real-time PCR.

### 2.12. Lactate Dehydrogenase Cytotoxicity Detection

We utilized LDH kits (C0017, Beyotime, Haimen, China) to measure LDH release. We plated an appropriate amount of cells into a 96 well cell culture plate, including cell-free culture medium wells (background blank), untreated control cell wells (sample control), untreated cell wells for calculation (maximum enzyme activity), and drug-treated cell wells (drug-treated). One hour before the detection time, the cell culture plate was removed from the cell culture box and the LDH release reagent added to the maximum enzyme activity wells, in an amount of 10% of the original culture medium volume. After reaching the scheduled time, the cell culture plate was centrifuged at 400× *g* for 5 min. Then, 60 μL of LDH detection working solution was added to each well, mixed, and incubated at room temperature in the dark for 30 min. Then, we measured the absorbance at 490 nm and used any wavelength of 600 nm or greater as the reference wavelength for dual-wavelength measurement. The absorbance for each group obtained should be subtracted by the absorbance from the background control wells. The percentage of cytotoxicity (%) = (absorbance of sample control/drug treated)/(absorbance to maximum enzymatic activity − absorbance of sample control) × 100.

### 2.13. Lipid Peroxidation MDA Detection

The level of MDA was measured by an MDA assay kit (S0131M, Beyotime, Haimen, China). After tissue and cell homogenization, samples were centrifuged at 12,000× *g* for 10 min and the supernatant collected for subsequent measurement. A suitable amount of TBA was weighed and added into a 0.37% TBA stock solution using the TBA-reagent solution. The prepared TBA stock solution was stored at room temperature out of light. The MDA detection working solution was formulated as TBA diluent/TBA stock/antioxidant = 150:50:3. Appropriate standards were diluted to concentrations of 1, 2, 5, 10, 20, and 50 µM for subsequent standard curve preparation. Then, 100 µL of PBS control, standard, and samples were added to each 1.5 mL EP tube, followed by the addition of 200 µL MDA detection working solution. The mixture was then heated at 100 °C for 15 min, cooled in a water bath to room temperature, and centrifuged at 1000× *g* for 10 min at room temperature. Next, 200 µL supernatant was added to a 96-well plate, and absorbance was measured at 532 nm using a microplate reader. A dual-wavelength measurement was set with 450 nm as the reference wavelength. After determining the protein concentration using the BCA kit (P0009, Beyotime, Haimen, China), the MDA content in the original samples was expressed based on the protein content as umol/mg protein/tissue.

### 2.14. Measurement of Mitochondrial Membrane Potential (ΔΨm)

The measurement of ΔΨm was based on the JC-1 fluorescent probe (PJC-110; Promotor Biological Co., Ltd., Hangzhou, China). AC16 cells were incubated with JC-1 working solution for 30 min at 37 °C following treatments. Subsequently, the cells were washed at least two times with PBS and resuspended with cell culture medium. The fluorescence signals of JC-1 aggregates (red, 525/590 nm) and JC-1 monomers (green, 485/530 nm) were detected with an MShot fluorescence microscope (Wuhan, China). The ratio of the red/green fluorescence intensity represents the degree of mitochondrial damage.

### 2.15. Echocardiogram

After completing the model, the mice underwent chest hair removal, followed by anesthesia induction using the gas anesthesia machine. Cardiac ultrasound imaging was then performed using the VINNO6 high-resolution imaging system (VINNO Corporation, Suzhou, China), with anesthesia maintained (*n* = 10). Echocardiography was conducted to assess cardiac function by recording the left ventricular end-diastolic volume (LEDV) and left ventricular end-systolic volume (LESV).

### 2.16. HE, Masson Staining, and Immunohistochemistry

After animals were sacrificed, the tissues were fixed with 4% formaldehyde. The formalin-fixed tissue was embedded in paraffin and sectioned for further analysis. Masson staining was used for collagen deposition, and HE staining was used to observe the structure of tissues. As for immunohistochemistry, the fixed paraffin sections were incubated with the primary antibody overnight at 4 °C. After washing three times with PBS, the sections were incubated with secondary antibody for 1 h. Then, after washing three times, they were scanned with a brightfield scanner and observed using the NDP.view 2 software 2.9.29. The primary antibodies used in this study targeted endogenous ACAA2 (1:300, A15778, ABclonal, Wuhan, China).

### 2.17. Oil Red O Staining

Tissues were prepared with a thickness of 4–8 μm and dried for 15–30 min at room temperature. The sections were fixed with 4% formaldehyde for 10 min, followed by three washes with TBST. The Oil Red O stock solution was diluted with distilled water at a 3:2 ratio and incubated at room temperature in the dark for 20–30 min. The sections were washed three times with TBST and counterstained with hematoxylin, and the slides were mounted using 50% glycerol. After scanning with a brightfield scanner, the lipid droplet aggregation was observed using the NDP.view 2 software 2.9.29.

### 2.18. Statistical Analysis

All data were assessed for normality, and then, parametric or non-parametric tests were employed for data analysis, as appropriate. The unpaired two-tailed *t*-test was used to compare data between two groups and one-way ANOVA with Sidak’s correction for multiple testing to compare data between more than two groups. The exact test used for each experiment is noted in the figure legends. Data are expressed as mean ± SEM. Statistical significance was considered when *p* < 0.05. * *p* < 0.05; ** *p* < 0.01; *** *p* < 0.001; **** *p* < 0.0001; ns means not significant. All statistical analysis was performed using GraphPad Prism 10.1.1.

## 3. Results

### 3.1. Uremic Myocardial Metabolic Abnormalities Linked to Fatty Acid β-Oxidation Pathway

**Figure 1:**
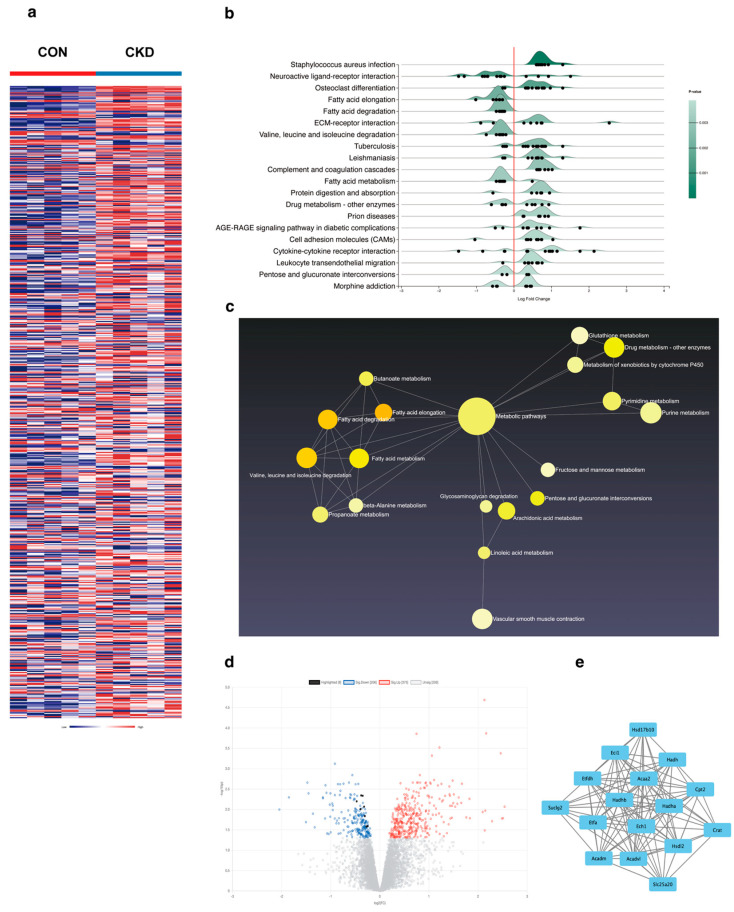
GEO database analysis shows that metabolic changes in cardiac function in mice with renal insufficiency are closely related to fatty acid β-oxidation. (**a**) Heatmap of significantly differentially expressed genes between CKD mice and the control group; (**b**) ridge plot of enriched pathways for differentially expressed genes; (**c**) 2D schematic diagram of metabolic pathways enriched in differentially expressed genes (the larger the circle, the more the genes are enriched); (**d**) volcano plot of differentially expressed genes (black dots represent genes related to fatty acid β-oxidation); (**e**) protein–protein interaction PPI network of fatty acid β-oxidation-related proteins.

We utilized the Gene Expression Omnibus (GEO) database and retrieved the GSE106385 dataset by searching for the keywords “renal insufficiency” and “heart failure”. Differential gene expression analysis (Figure 1a) and enrichment analysis (Figure 1b) were performed. Our findings indicated that the pathways related to cardiac function in mice with renal impairment were predominantly altered in metabolic pathways (Figure 1c), while genes associated with fatty acid metabolism were the most affected. Specifically, the fatty acid β-oxidation pathway was significantly impacted (Figure 1d), suggesting a dysregulation of cardiac lipid metabolism in the context of renal insufficiency, although the specific targets remained unclear. Subsequently, after standardization and normalization of the data, we employed STRING and Cytoscape software 3.9.1 to construct protein–protein interaction (PPI) networks related to fatty acid metabolism. The results highlighted the critical role of the fatty acid β-oxidation pathway in these interactions (Figure 1e).

### 3.2. ACAA2 Decreased with Lipid Accumulation in Cardiomyocytes Induced by Indoxyl Sulfate

**Figure 2:**
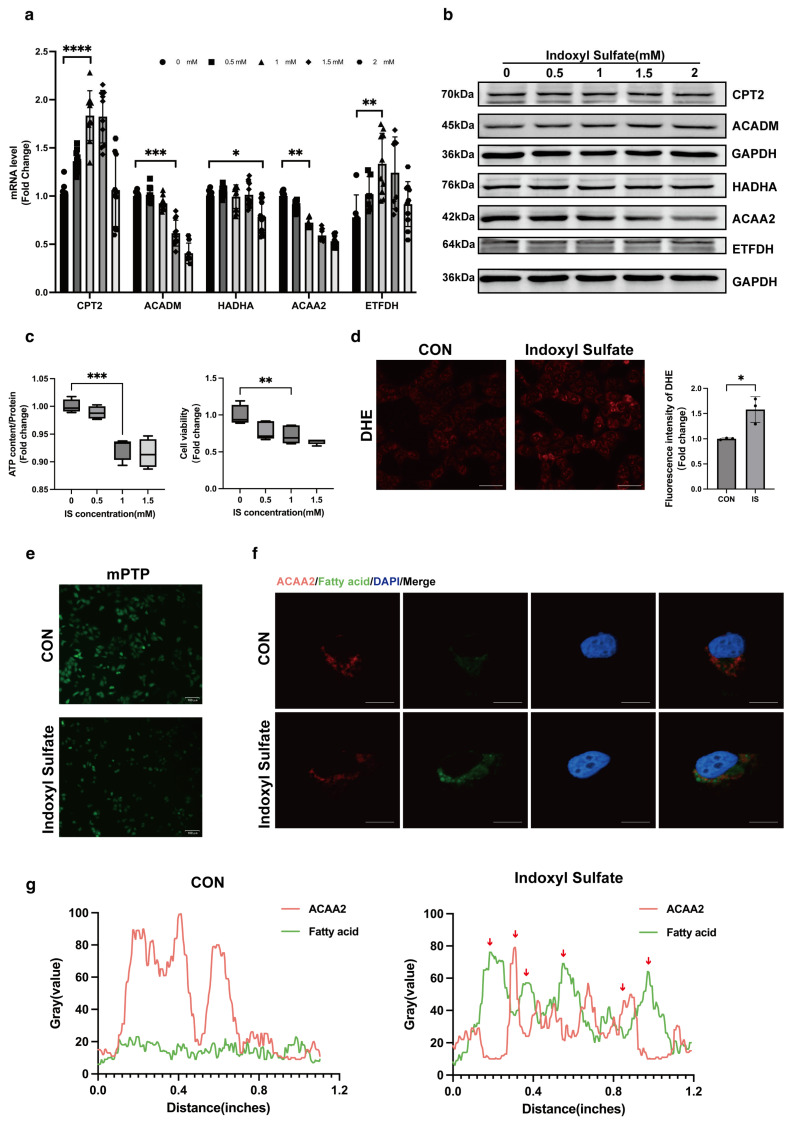
Indoxyl sulfate reduces ACAA2 expression in cardiomyocytes and promotes cardiac lipid peroxidation. (**a**) RNA expression levels in AC16 cells treated with different concentrations of indoxyl sulfate, 0–2 mM, *n* = 9; * *p* < 0.05, ** *p* < 0.01, *** *p* < 0.001, and **** *p* < 0.0001, two-way ANOVA. (**b**) Protein expression levels in AC16 cells treated with different concentrations of indoxyl sulfate, 0–2 mM; (**c**) ATP levels and CCK8 in AC16 cells stimulated by indoxyl sulfate 1 mM, *n* = 4; ** *p* < 0.01 and *** *p* < 0.001, one-way ANOVA. (**d**) DHE staining of AC16 cells stimulated by indoxyl sulfate 1 mM and quantitative analysis, scale bar: 50 μm, *n* = 3; * *p* < 0.05, *t*-tests. (**e**) Mitochondrial permeability transition pore in AC16 cells stimulated by indoxyl sulfate 1 mM, scale bar: 100 μm; CON: control group without any stimulation. (**f**,**g**) Immunofluorescence co-staining of ACAA2 protein and fatty acid probe BODIPY in AC16 cells after indoxyl sulfate 1 mM stimulation (**f**) and quantitative analysis (**g**) (red arrows: colocalization of ACAA2 protein and lipid droplets), scale bar: 50 μm. The original Western Blot Figures can be found in Supplementary Materials.

To validate the protein–protein interaction (PPI) network genes enriched from our analysis, we examined their expression in AC16 cardiomyocytes. Cardiomyocytes were stimulated with various concentrations (0–2 mM) of indoxyl sulfate, and after 24 h of serum starvation, proteins and RNA were collected. Interestingly, low concentrations of indoxyl sulfate increased the mRNA level of CPT2 and ETFDH, while only ACADM and ACAA2 showed a decreasing trend, suggesting that ACADM and ACAA2 proteins were more sensitive to stimulation (Figure 2a). Importantly, the Western blot results showed that only the ACAA2 protein exhibited a downward trend with increasing IS concentration (Figure 2b). And the ACAA2 protein, as a convergence point for fatty acid oxidation, the TCA cycle, and cholesterol synthesis, captured our interest. Subsequently, under the stimulation of indoxyl sulfate, cell viability and CCK8 and ATP content in AC16 cells both decreased (Figure 2c). Under 1 mM IS stimulation, cell viability in AC16 cardiomyocytes decreased slowly, while ATP levels dropped significantly. This suggested that 1 mM IS is sufficient to inflict damage on AC16 cells, and thus, 1 mM IS was used in subsequent experiments. Simultaneously, we conducted DHE staining and mPTP staining, revealing that indoxyl sulfate elevated oxidative stress levels within AC16 cells and promoted the opening of the mitochondrial permeability transition pore, indicating that indoxyl sulfate impairs mitochondrial function in cardiomyocytes (Figure 2d,e). ACAA2 is a mitochondrial protein, and we next performed co-staining of ACAA2 with the green fluorescent fatty acid probe BODIPY. We observed a significant reduction in ACAA2 protein and a notable increase in green fluorescence aggregation under indoxyl sulfate stimulation (Figure 2f). Additionally, line analysis of the green fluorescence aggregation sites revealed a high degree of overlap with the regions of reduced ACAA2 protein (Figure 2g), suggesting a close correlation between the decrease in ACAA2 and the process of fatty acid oxidation as well as a strong association between the reduction in ACAA2 and the accumulation of lipid intermediates. Combined with the important function of ACAA2 in the last step of fatty acid β oxidation, the above findings suggest that the reduction in ACAA2 hinders the process of fatty acid oxidation, causing the accumulation of lipid intermediates and fatty acids, thereby increasing intracellular oxidative stress levels and damaging mitochondrial function.

### 3.3. Knockdown and Overexpression ACAA2 Affect Mitochondrial Function and Oxidative Stress in Cardiomyocytes

**Figure 3:**
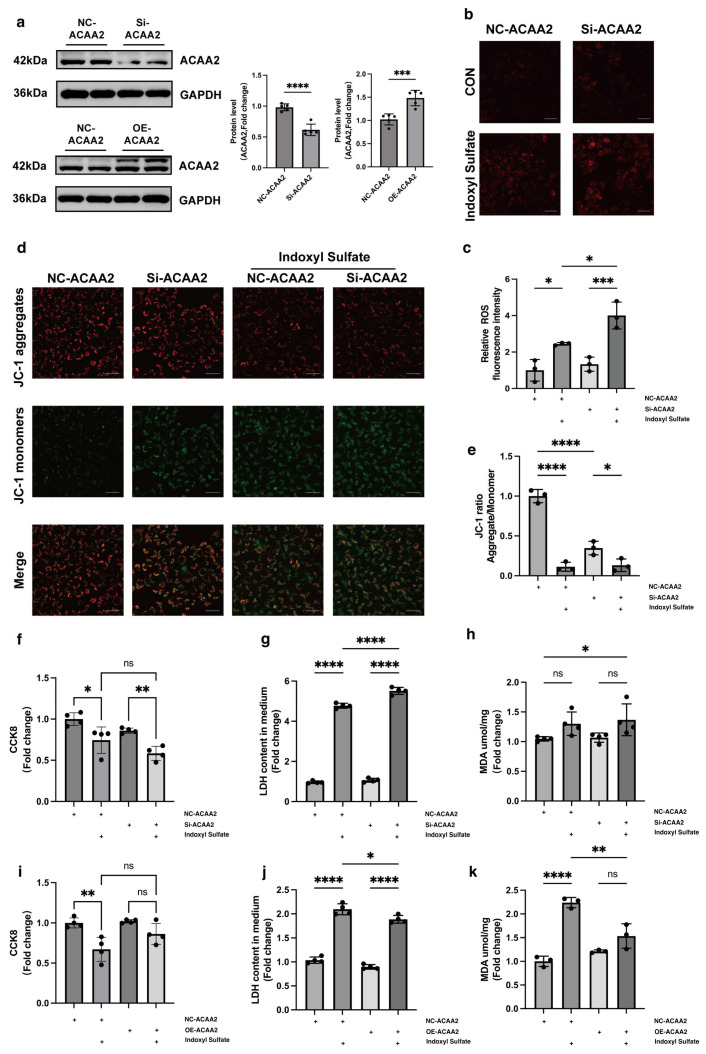
Knockdown of ACAA2 protein promotes mitochondrial damage and oxidative stress in AC16 cells, while overexpression alleviates these effects. (**a**) Protein expression levels of ACAA2 after knockdown and overexpression, *n* = 5. (**b**–**e**) DHE staining (**b**) and corresponding quantitative analysis (**c**), JC-1 staining, (**d**) and corresponding quantitative analysis (**e**) of AC16 cells after ACAA2 knockdown under indoxyl sulfate stimulation, scale bar: 50 μm, *n* = 3; * *p* < 0.05, *** *p* < 0.001, and **** *p* < 0.0001, one-way ANOVA. (**f**–**h**) Cell viability (**f**), lactate dehydrogenase (LDH) levels (**g**), and MDA levels (**h**) of AC16 cells after ACAA2 knockdown under indoxyl sulfate stimulation, *n* = 4; * *p* < 0.05, ** *p <* 0.01, **** *p* < 0.0001, and ns: not significant, one-way ANOVA. (**i**–**k**) Cell viability (**i**), lactate dehydrogenase LDH levels (**j**), and MDA levels (**k**) of AC16 cells after ACAA2 overexpression under indoxyl sulfate stimulation, *n* = 3–4; * *p* < 0.05, ** *p* < 0.01, **** *p* < 0.0001, and ns: not significant. “+” means the operation in the same row was performed. The original Western Blot Figures can be found in Supplementary Materials.

We constructed plasmids for ACAA2 overexpression and knockdown and transfected them into AC16 cells to verify the expression effects (Figure 3a, Supplementary Figure S1b). In AC16 cells, after knocking down ACAA2, we observed through DHE staining that although the intracellular ROS level did not significantly increase after simply knocking down ACAA2, under the stimulation of indoxyl sulfate, knocking down ACAA2 promoted an increase in intracellular ROS levels. This suggested that ACAA2 has a protective effect on cardiomyocytes under the stimulation of urea toxins (Figure 3b,c). To further elucidate the protective role of ACAA2 on mitochondria in cardiomyocytes under the stimulation of indoxyl sulfate, we used JC-1 staining to assess mitochondrial membrane potential levels. The experiment revealed that the mitochondrial membrane potential was significantly reduced when ACAA2 was solely knocked down, and the stimulation of indoxyl sulfate also significantly decreased the membrane potential levels in AC16 cells. This does not conflict but rather underscores the role of ACAA2 in maintaining mitochondrial function in AC16 cells (Figure 3d,e). CCK8, LDH, and MDA assays were conducted in AC16 cells. Although there was no significant decrease in the cell viability of si-ACAA2 stimulated by IS, there was a clear trend (Figure 3f). Under the stimulation of indoxyl sulfate with si-ACAA2, the release of lactate dehydrogenase from mitochondria further increased, and MDA indicated a further elevation in intracellular lipid peroxidation levels (Figure 3g,h). However, in AC16 cells with overexpression of ACAA2, under the stimulation of indoxyl sulfate, the trend of cell viability increased, albeit not significantly, while the release of mitochondrial lactate dehydrogenase significantly decreased, and the level of lipid peroxidation as measured by MDA also declined (Figure 3i–k). The above experiments indicated that knocking down ACAA2 exacerbated mitochondrial damage in AC16 cells induced by indoxyl sulfate, whereas overexpression of ACAA2 alleviated mitochondrial damage and lipid peroxidation under indoxyl sulfate stimulation, suggesting that ACAA2 had a protective effect on mitochondrial damage under indoxyl sulfate stimulation.

### 3.4. Knockdown FOXO4 Restores ACAA2 Protein Level and Alleviates Mitochondrial Dysfunction

**Figure 4:**
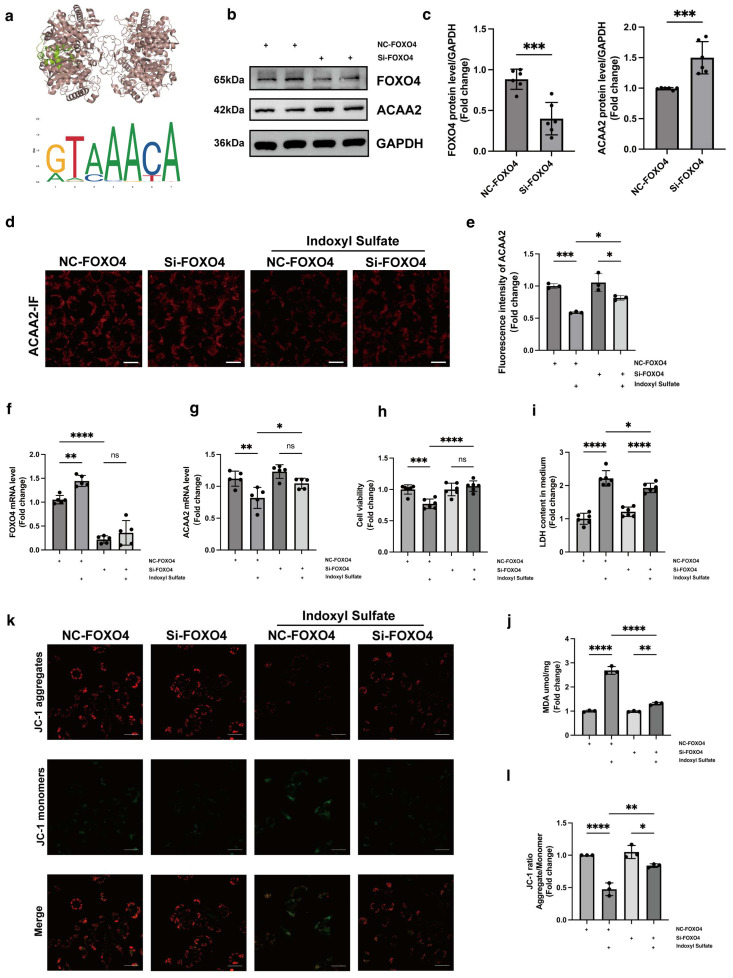
Knockdown of FOXO4 restores ACAA2 protein expression and alleviates mitochondrial damage. (**a**) Protein docking diagram of FOXO4 (green) and ACAA2 (red) and the transcription factor sequence motif from the JASPAR database for FOXO4. (**b**,**c**) Protein expression levels (**b**) after FOXO4 knockdown and quantitative analysis (**c**), *n* = 6. (**d**,**e**) Immunofluorescence expression of ACAA2. (**d**) and corresponding quantitative analysis (**e**) in AC16 cells after FOXO4 knockdown under indoxyl sulfate stimulation, scale bar: 50 μm, *n* = 3; * *p* < 0.05 and *** *p* < 0.001, one-way ANOVA. (**f**,**g**) mRNA levels of FOXO4 (**f**) and ACAA2 (**g**) in AC16 cells after FOXO4 knockdown under indoxyl sulfate stimulation, *n* = 5; * *p* < 0.05, ** *p <* 0.01, **** *p* < 0.0001, and ns: not significant, one-way ANOVA. (**h**–**j**) Cell viability (**h**), lactate dehydrogenase LDH levels (**i**), and MDA levels (**j**) in AC16 cells after FOXO4 knockdown under indoxyl sulfate stimulation, *n* = 6 and *n* = 3; * *p* < 0.05, ** *p <* 0.01, *** *p* < 0.001, **** *p* < 0.0001, and ns: not significant, one-way ANOVA. (**k**,**l**) JC-1 staining (**k**) and corresponding quantitative analysis (**l**) of AC16 cells after FOXO4 knockdown under indoxyl sulfate stimulation, scale bar: 50 μm, *n* = 3; * *p* < 0.05, ** *p* < 0.01, and **** *p* < 0.0001, one-way ANOVA. “+” means the operation in the same row was performed. The original Western Blot Figures can be found in Supplementary Materials.

Through the JASPAR database, we predicted that FOXO4 might be an upstream transcription factor of ACAA2, and we simulated the binding of FOXO4 and ACAA2 molecules using Pymol 2.5.5 and other software (Figure 4a, Supplementary Figure S1a). To our knowledge, FOXO4 as a transcriptional regulator upstream of ACAA2 has not been reported previously. Subsequently, we knocked down FOXO4 in AC16 cells and collected RNA for subsequent experiments. The knockdown efficiency of FOXO4 results indicated that the 003 sequence for FOXO4 knockdown could be used for subsequent experiments (Supplementary Figure S1b). Through Western blot experiment, the expression of ACAA2 protein in AC16 cells after FOXO4 knockdown was significantly elevated (Figure 4b,c). Subsequently, we added indoxyl sulfate into AC16 cells with si-FOXO4 to further observe changes of ACAA2 protein levels. Through immunofluorescence staining for ACAA2, we directly observed that the stimulation of indoxyl sulfate did not result in a reduction in ACAA2 protein despite the knockdown of FOXO4. This suggests that the activation of the transcription factor FOXO4 may inhibit the synthesis of ACAA2 protein (Figure 4d,e). In AC16 cells with si-FOXO4 stimulated by indoxyl sulfate, Rt-qPCR results showed that the FOXO4 mRNA level did not increase, while the ACAA2 mRNA level significantly increased compared to indoxyl sulfate stimulation alone. Consistent with previous immunofluorescence results, this suggests that the transcription factor FOXO4 regulates the downstream ACAA2 protein (Figure 4f,g). Then, mitochondrial function assays were conducted with si-FOXO4 in AC16 cells. We observed that under indoxyl sulfate stimulation, si-FOXO4 could restore cell viability (Figure 4h), reduce the release of mitochondrial lactate dehydrogenase (Figure 4i), and decrease the intracellular level of lipid peroxidation MDA level (Figure 4j). Additionally, JC-1 staining indicated that the reduction in mitochondrial membrane potential in AC16 cells stimulated by indoxyl sulfate could be reversed by si-FOXO4 (Figure 4k,l). These results indicate that FOXO4 knockdown in vitro effectively restored ACAA2 expression and protected cardiomyocytes by reducing mitochondrial damage and lipid peroxidative stress.

### 3.5. CSNO Alleviates Uremic Toxin-Induced Mitochondrial Dysfunction and Lipid Peroxidation in Cardiomyocytes In Vitro

**Figure 5:**
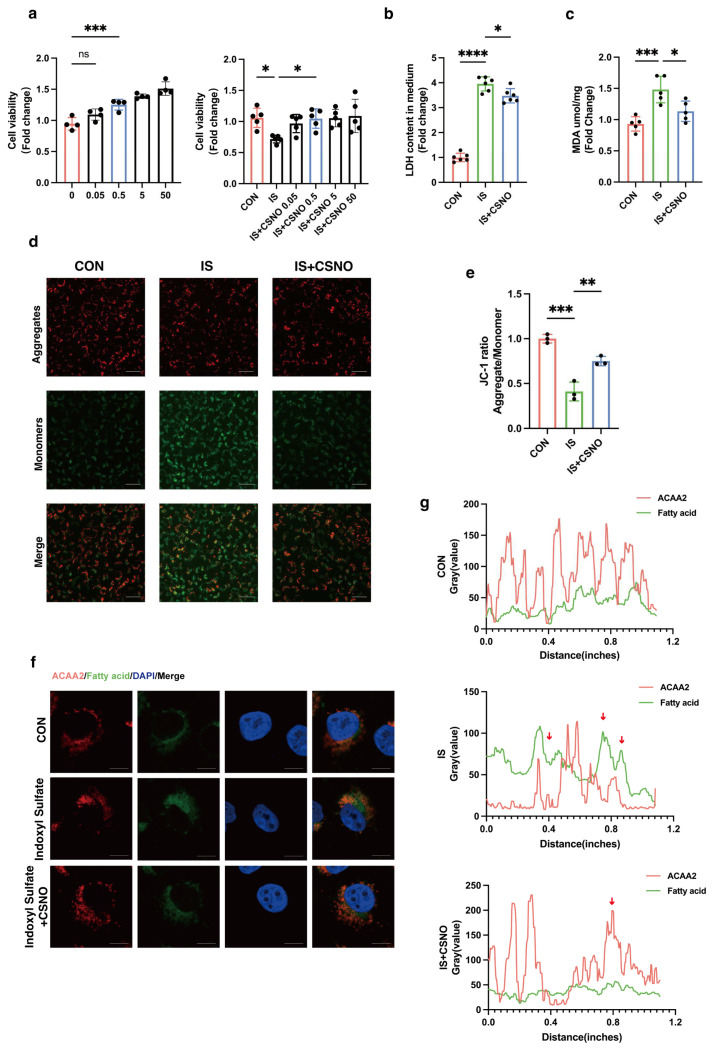
CSNO alleviates mitochondrial damage and lipid peroxidation in cardiomyocytes induced by uremic toxins. (**a**) Cell viability of AC16 cells treated with different concentrations of CSNO (0.05–100 μM) with or without indoxyl sulfate, *n* = 4–5; * *p* < 0.05 and *** *p* < 0.001, ns: not significant, one-way ANOVA. (**b**) LDH levels in AC16 cells treated with CSNO under indoxyl sulfate stimulation, *n* = 6; * *p* < 0.05 and **** *p* < 0.0001, one-way ANOVA. (**c**) MDA levels in AC16 cells treated with CSNO under indoxyl sulfate stimulation, *n* = 5. (**d**,**e**) JC-1 staining (**d**) and corresponding quantitative analysis (**e**) of AC16 cells treated with CSNO under indoxyl sulfate stimulation, scale bar: 50 μm, *n* = 3; ** *p* < 0.01, and *** *p* < 0.001, one-way ANOVA. (**f**,**g**) Immunofluorescence co-staining (**f**) and quantitative analysis (**g**) of ACAA2 protein and the fatty acid probe BODIPY in AC16 cells treated with CSNO under indoxyl sulfate stimulation (red arrows: colocalization of ACAA2 protein and lipid droplets), scale bar: 50 μm.

Our previous studies demonstrated that CSNO can improve glucose metabolism disorders [24], and we found that CSNO also has an inhibitory effect on lipid peroxidation under urea toxin stimulation. Firstly, we conducted a CSNO concentration-dependent experiment on AC16 cells, observing that the promotion of cell proliferation in AC16 cells by CSNO began to significantly increase at 0.5 µM. Subsequently, we added indoxyl sulfate to the mixture and continued to use different concentrations of CSNO. The results again showed that 0.5 µM of CSNO was sufficient to reverse the activity damage to AC16 cells caused by indoxyl sulfate. Hence, experiments were conducted using a CSNO concentration of 0.5 µM (Figure 5a). We then proceeded to investigate how CSNO could enhance the cellular activity of indoxyl sulfate in AC16 cells. CSNO reduced mitochondrial damage in AC16 cells induced by IS not only by decreasing the release of LDH from mitochondria (Figure 5b) but also by restoring the mitochondrial membrane potential (Figure 5d,e). Additionally, the recovery of MDA levels suggested that CSNO significantly reduced lipid peroxidation level in AC16 cells (Figure 5c). We then stained AC16 cells with the fatty acid probe BODIPY and found that the CSNO group significantly reduced the formation of lipid intermediates compared to the IS group (Figure 5f). Co-staining ACAA2 protein with the fatty acid probe revealed that the formation of lipid intermediates in the CSNO group overlapped with the fluorescent absent regions of ACAA2 protein (Figure 5g). Previous results indicated that ACAA2 protein is crucial for antioxidant stress in cardiomyocytes, and CSNO could restore ACAA2 expression, improve mitochondrial dysfunction in cardiomyocytes caused by IS, and alleviate lipid peroxidation.

### 3.6. CSNO Ameliorates Uremic Toxin-Induced Mitochondrial Damage and Lipid Peroxidation in Cardiomyocytes Through FOXO4–ACAA2 Axis In Vitro

**Figure 6:**
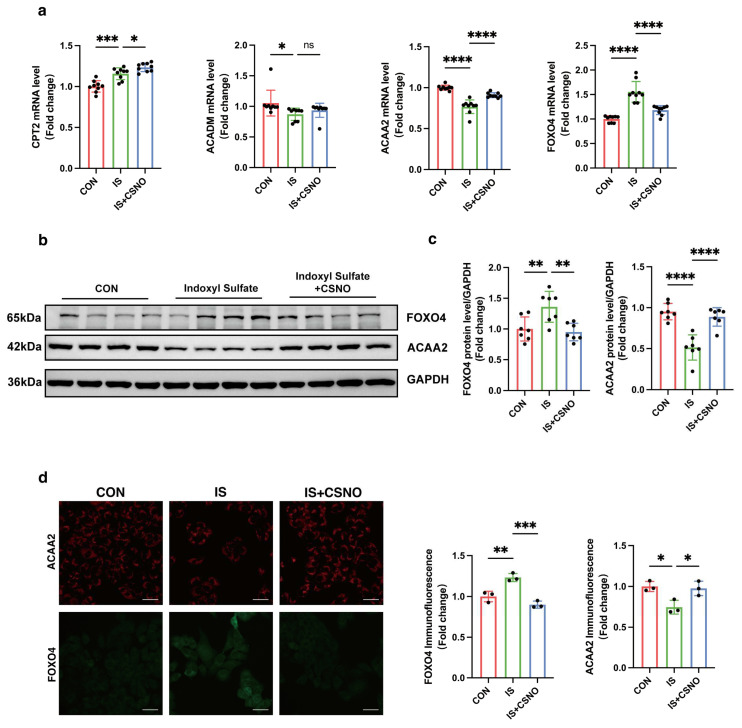
CSNO participates in the regulation of the FOXO4–ACAA2 axis. (**a**) mRNA levels in AC16 cells treated with CSNO under indoxyl sulfate stimulation, *n* = 9, one-way ANOVA. (**b**,**c**) Protein levels of ACAA2 and FOXO4 (**b**) and corresponding quantitative analysis (**c**) in AC16 cells treated with CSNO under indoxyl sulfate stimulation, *n* = 7. (**d**) Immunofluorescence levels of ACAA2 and FOXO4 (**d**) and corresponding quantitative analysis in AC16 cells treated with CSNO under indoxyl sulfate stimulation, scale bar: 50 μm, *n* = 3; * *p* < 0.05, ** *p* < 0.01, *** *p* < 0.001, **** *p* < 0.0001 and ns: no significant, one-way ANOVA. The original Western Blot Figures can be found in Supplementary Materials.

Previously, we demonstrated in AC16 cells that CSNO could alleviate the mitochondrial damage and lipid oxidative stress in cardiomyocytes under uremic toxin stimulation. This prompted us to explore the mechanism of CSNO. We collected RNA samples from AC16 cells to examine the genes involved in lipid metabolism. Given the interacting proteins identified in the GEO database that implicate ACAA2 in lipid metabolism progression, we initially assessed the changes in these genes following CSNO treatment (Supplementary Table S2). We observed that, following CSNO treatment, lipid transport-associated proteins such as CPT2 experienced further upregulation, while proteins critical for fatty acid β oxidation, including ACADM, ECH1, and HADHA, remained largely unrecovered (Figure 6a, Supplementary Figure S1c). CSNO appeared to uniquely restore ACAA2 protein levels, suggesting a specific effect of CSNO on ACAA2. Furthermore, we assessed the transcription factor FOXO4, known to regulate ACAA2 expression, and found that CSNO diminished the suppressive effect of FOXO4 on ACAA2 (Figure 6a). This encouraging result led us to subsequently demonstrate, through both Western blot (Figure 6b,c) and immunofluorescence (Figure 6d), the changes in ACAA2 and FOXO4 proteins under CSNO treatment. Consistently, under IS stimulation in AC16 cells, activation of FOXO4 inhibited the expression of ACAA2 protein. The decrease in ACAA2 led to lipid peroxidation in cardiomyocytes, causing mitochondrial injury. However, CSNO treatment could reduce the excessive activation of FOXO4, thereby restoring ACAA2 expression and alleviating mitochondrial damage caused by lipid oxidative stress.

### 3.7. CSNO Nebulization Improves Cardiac Function in Mice with Renal Insufficiency In Vivo

**Figure 7:**
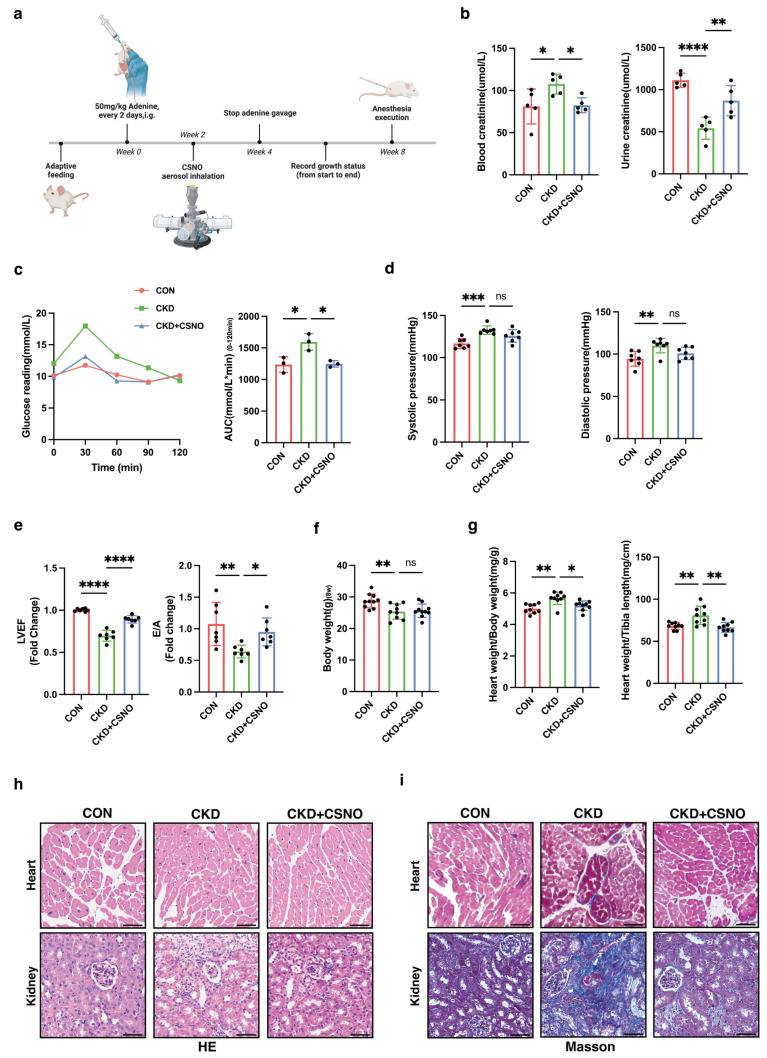
CSNO inhalation improves cardiac function in mice with renal insufficiency. (**a**) Schematic diagram of the mouse model construction and treatment protocol. (**b**) Blood and urine creatinine levels in mice, *n* = 5, * *p* < 0.05, ** *p* < 0.01, **** *p* < 0.0001, one-way ANOVA. (**c**) OGTT glucose tolerance levels and area under the curve AUC analysis in mice, *n* = 3. (**d**) Systolic and diastolic blood pressure in mice, *n* = 7, ** *p* < 0.01, *** *p* < 0.001, and ns: no significant, one-way ANOVA. (**e**) Left ventricular ejection fraction LVEF and E/A ratio of diastolic function in mice, *n* = 7. (**f**) Body weight of mice at the end of 8 weeks, *n* = 9. (**g**) Heart-to-body weight ratio and heart-to-tibia length ratio in mice, *n* = 9. (**h**,**i**) HE staining (**h**) and Masson staining (**i**) of mouse heart and kidney tissues, scale bar: 50 μm.

Subsequently, we conducted validation experiments using C57BL/6 mice. The mice were divided into three groups: control group, CKD group, and CKD + CSNO inhalation group, and the experiments lasted for 8 weeks. By the end of the 8th week, mice were induced into anesthesia, underwent echocardiographic assessment of cardiac function, and were then euthanized to collect samples for further experiments (Figure 7a). Firstly, we detected the creatinine levels in blood and urine samples, finding significantly elevated serum creatinine and decreased urinary creatinine in the CKD group, indicating the successful establishment of the renal dysfunction model. Meanwhile, the CSNO group showed a marked reduction in circulating creatinine and enhanced excretion of creatinine, suggesting that nebulized CSNO therapy could improve renal dysfunction (Figure 7b). At the same time, consistent with cell experiments, we measured the concentration of IS in blood samples (Supplementary Figure S1d). Additionally, the OGTT results suggested that treatment with CSNO aided in reducing blood glucose levels in mice with impaired renal function (Figure 7c). The tail-cuff blood pressure results indicated that both systolic and diastolic blood pressure showed varying degrees of elevation in the CKD group, while CSNO treatment, though not significantly reducing blood pressure, showed a downward trend in both (Figure 7d). Although CSNO did not significantly restore blood pressure, it exerted significant effects on the recovery of left ventricular systolic function (Figure 7e), indicating that the restoration of CSNO in cardiac function was not achieved solely through reducing blood pressure but involved other mechanisms. By measuring the body weight of mice at the end of the 8th week, we found that CSNO treatment did not restore the weight loss in mice under renal insufficiency (Figure 7f, Supplementary Figure S1e). However, by measuring the heart-to-body weight ratio and heart-to-tibia length ratio in mice (Figure 7g), we found that both were reduced in the CSNO group compared to the CKD group, indicating that CSNO treatment alleviated cardiac hypertrophy in mice. Subsequently, we performed HE and MASSON staining (Figure 7h,i) on the heart and kidney tissues of mice and found that the nebulized inhalation of CSNO alleviated myocardial hypertrophy and alleviated renal fibrosis to a certain extent. Furthermore, upon Oil Red O staining of mouse heart and kidney tissues, consistent results were obtained (Supplementary Figure S2). The results above indicate that inhalation of CSNO could mitigate renal fibrosis in cases of renal insufficiency and restore cardiac function by alleviating myocardial hypertrophy independent of blood pressure reduction.

### 3.8. CSNO Improves Cardiac Function in Mice with Renal Insufficiency via the FOXO4–ACAA2 Axis In Vivo

**Figure 8:**
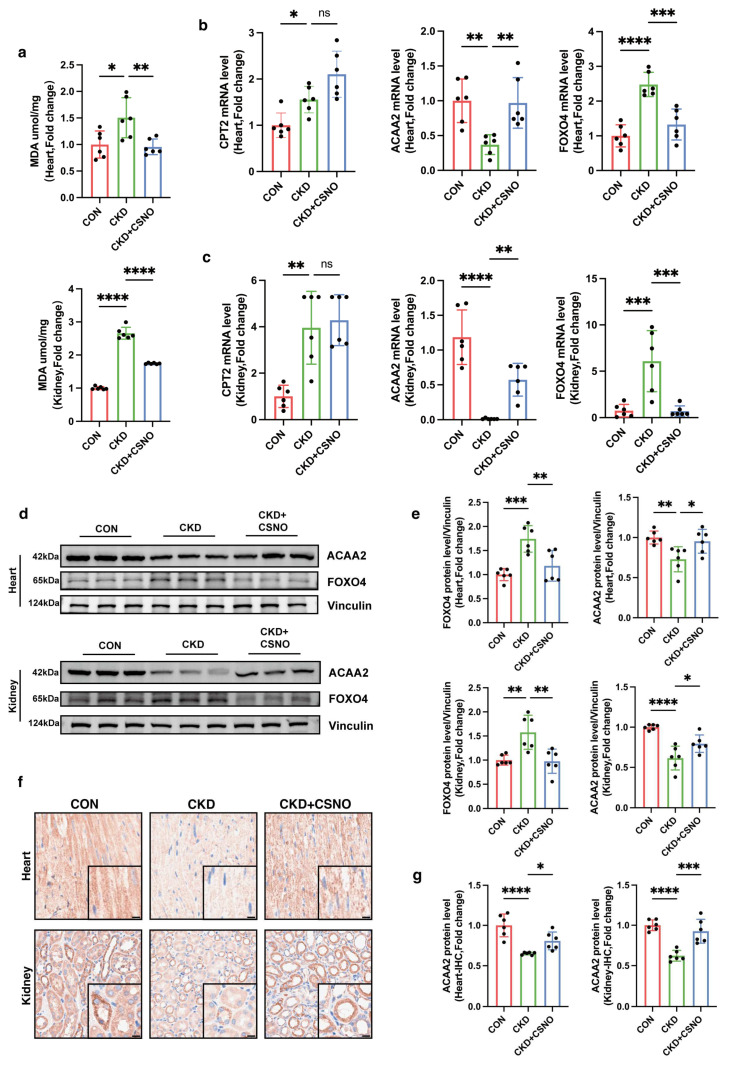
CSNO inhalation regulates the FOXO4–ACAA2 axis in vivo and reduces cardiac and renal lipid peroxidation. (**a**) MDA levels in the heart and kidney of mice, *n* = 6. (**b**,**c**) mRNA levels in the heart (**b**) and kidney (**c**) of mice, *n* = 6. (**d**,**e**) Protein expression levels in the heart and kidney of mice (**d**) and corresponding quantitative analysis (**e**), *n* = 6. (**f**,**g**) Immunohistochemistry of ACAA2 in the heart and kidney of mice (**f**) and corresponding quantitative analysis (**g**), black small box: local enlargement, scale bar: 10 μm, *n* = 6; * *p* < 0.05, ** *p* < 0.01, *** *p* < 0.001, and **** *p* < 0.0001, ns: not significant, one-way ANOVA. The original Western Blot Figures can be found in Supplementary Materials.

We separately tested MDA levels in heart and kidney tissues to verify whether CSNO could alleviate lipid peroxidative stress. The results indicated that both heart and kidney under renal dysfunction showed varying degrees of lipid peroxidation, which was alleviated after nebulized inhalation of CSNO (Figure 8a). To verify the regulatory relationship within the FOXO4–ACAA2 axis, we collected RNA samples from heart and kidney tissues and performed RT-qPCR (Figure 8b,c). CPT2, a lipid transporter in the heart and kidney, significantly increased during CKD modeling, indicating a promotion in fatty acid transport, while proteins related to fatty acid β oxidation, such as ACAA2 and ACADM (Supplementary Figure S1f), showed a decrease. This suggests that under external conditions of kidney dysfunction, both heart and kidney organs exhibit varying degrees of fatty acid metabolism decompensation in mice. Following nebulized inhalation of CSNO, though CPT2 further increased, there was a significant recovery in the ACAA2 protein, indicating that CSNO specifically restored ACAA2 protein, driving lipid metabolism from decompensation to compensation. The upstream transcription factor FOXO4 also showed consistent changes. Subsequent Western blot (Figure 8d,e) and immunohistochemical analysis (Figure 8f,g) of proteins related to the FOXO4–ACAA2 axis revealed that ACAA2 showed varying degrees of significant reduction in heart and kidney tissues under CKD modeling, which CSNO could reverse. This indicates that CSNO exerted protective effects on heart and kidney organs under kidney dysfunction through the FOXO4–ACAA2 axis, resisting lipid peroxidation and maintaining the normal functioning of heart and kidney tissues.

## 4. Discussion

Cardiorenal syndrome refers to the complex interplay between the heart and kidneys. Studies have shown that cardiac remodeling in patients with chronic kidney disease (CKD) is closely associated with metabolic abnormalities in cardiomyocytes, particularly alterations in energy metabolism [28]. Moreover, uremic cardiomyopathy is a common cardiac complication in CKD patients, characterized by myocardial hypertrophy and fibrosis and often accompanied by left ventricular dysfunction. The pathophysiological mechanisms of uremic cardiomyopathy are intricate, involving factors such as electrolyte imbalance, activation of the renin–angiotensin system, and hyperactivity of the sympathetic nervous system. Clinically, despite improvements in adverse factors such as high circulatory load, heart function in patients with both cardiac and renal insufficiency may continue to deteriorate. This observation suggests that renal dysfunction may impair cardiac function through mechanisms other than hemodynamic overload. Therefore, investigating the mechanisms underlying non-hemodynamic cardiac injury in renal dysfunction and identifying effective interventions are of critical importance. Against this backdrop, our study focused on the elevated levels of uremic toxins in patients with renal dysfunction, particularly indoxyl sulfate IS.

The heart is a highly metabolically active organ that primarily relies on fatty acid oxidation for energy supply. When the uptake of fatty acids exceeds the oxidative capacity of cardiomyocytes, unmetabolized fatty acids accumulate within the cells, forming intermediates such as triglycerides, diacylglycerol, and ceramides [29,30]. These intermediates not only directly impact the physiological functions of cardiomyocytes but also activate various intracellular signaling pathways, inducing oxidative stress, inflammatory responses, and apoptosis, thereby exacerbating myocardial injury. Studies have demonstrated that in patients with metabolic disorders such as obesity, type 2 diabetes, and hyperlipidemia, the capacity for myocardial fatty acid oxidation is significantly impaired, leading to abnormal lipid accumulation in the myocardium and resulting in lipid peroxidation [31]. Through GEO data analysis, we found that fatty acid β-oxidation-related proteins in the heart are markedly downregulated under the context of renal dysfunction, with ACAA2 and other related fatty acid metabolic factors playing a critical role in fatty acid metabolism. This suggests that fatty acid oxidation is unable to provide sufficient energy support for cardiomyocytes under renal insufficiency. In AC16 cardiomyocytes stimulated with 1 mM indoxyl sulfate IS, we observed increased cell death rates, decreased mitochondrial ATP levels, elevated mitochondrial oxidative stress, and opening of the mitochondrial permeability transition pore (mPTP), indicating that moderate uremic toxin exposure impairs cardiomyocyte and mitochondrial function. Interestingly, colocalization of ACAA2 protein with lipid droplets showed that indoxyl sulfate reduces ACAA2 expression while increasing lipid droplet accumulation. In addition, IS-stimulated cardiomyocytes exhibited increased mRNA levels of the fatty acid transporter CPT2 and electron transfer protein ETFDH, while β-oxidation-related proteins such as ACADM, Ech1, and ACAA2 were significantly decreased. These findings indicated that, although fatty acid β-oxidation is diminished, fatty acid uptake by cardiomyocytes remains largely unaffected. This suggests that while the myocardium continuously takes up fatty acids for oxidation, the metabolic “fuel factory” fails to operate effectively, resulting in the accumulation of lipid metabolic intermediates within the mitochondria, triggering a cascade of oxidative stress reactions, including lipid peroxidation. However, it is worth noting that there are asymmetric changes in the protein levels and mRNA levels of CPT2, ACADM, and ETFDH. Protein level expression is not only associated with transcription and translation processes but also related to protein degradation. Proteins may be rapidly degraded by the ubiquitin–proteasome system or the autophagy pathway, resulting in lower protein levels despite high mRNA levels. The mechanisms underlying this phenomenon remain to be explored. ACAA2, an acyl-CoA acyltransferase, is involved in the final step of fatty acid β-oxidation and exerts a protective role in various diseases [32,33]. Given this, we questioned whether modulation of ACAA2 might alleviate myocardial injury in renal dysfunction. Therefore, we knocked down and overexpressed ACAA2. Interestingly, simple knockdown of ACAA2 in AC16 cardiomyocytes did not significantly affect ROS levels or LDH in experiments, which suggests that knockdown of ACAA2 alone has limited effects on the accumulation of intracellular oxygen radicals, making it hard to trigger apoptosis or necrosis. However, when stimulating AC16 cells with IS, si-ACAA2 further amplified the toxic effects on cardiomyocytes, which suggests that ACAA2 protein is indispensable in protecting cardiomyocytes from damage induced by urea toxin stimulation. The decline in mitochondrial membrane potential with addition of si-ACAA2 indicated an early-stage damage, suggesting an acute temporal impact of si-ACAA2 on cardiomyocytes. In contrast, simply overexpressing ACAA2 did not significantly restore LDH and MDA levels in AC16 cells. However, under the external stimulus of IS, LDH and MDA levels significantly decreased, indicating that the underlying mechanisms require further investigation. Given that ACAA2 differs from other matrix-associated mitochondrial proteins by possessing a non-cleavable N-terminus, it may exhibit distinct functionalities under certain specific conditions. These results suggest that overexpression of ACAA2 facilitates the management of excess fatty acids within cardiomyocytes, reducing intracellular lipid peroxidation.

Having established the crucial role of ACAA2 in fatty acid β-oxidation, we further utilized the JASPAR database to predict upstream transcription factors potentially regulating ACAA2. Among these, FOXO4 emerged as a likely candidate. FOXO4, a member of the forkhead box transcription factor family, has received increasing attention in cancer research in recent years [34]. For instance, the hypoxia-induced FOXO4/LDHA axis was found to play a pivotal role in regulating glycolysis and tumor progression in gastric cancer [35]. We hypothesized that FOXO4 might exert transcriptional control over ACAA2, and to test this, we knocked down FOXO4 in AC16 cardiomyocytes, followed by stimulation with indoxyl sulfate. From multiple perspectives, including RNA and protein expression levels, we found that FOXO4 knockdown partially restored the reduction in ACAA2 protein levels induced by indoxyl sulfate. Furthermore, knocking down FOXO4 mitigated the effects of indoxyl sulfate on cardiomyocyte apoptosis, mitochondrial lactate dehydrogenase release, mitochondrial membrane potential, and lipid peroxidation levels. In summary, FOXO4 knockdown improved the oxidative stress and mitochondrial dysfunction in cardiomyocytes induced by indoxyl sulfate, partly through the restoration of ACAA2 expression. However, the transcriptional regulation of ACAA2 by FOXO4 did not appear to be highly specific. Our subsequent unpublished results suggest that the regulatory factors controlling ACAA2 expression are multifaceted and complex, extending beyond transcription factors and involving post-translational modifications. Nonetheless, it is unequivocal that FOXO4 is involved in the regulation of ACAA2 expression. However, further studies are needed to clarify how FOXO4 specifically influences lipid metabolism in cardiomyocytes through ACAA2.

Previous studies have demonstrated that CSNO can improve cardiac function in diabetic cardiomyopathy and restore cardiomyocyte viability [24]. In the cardiovascular system, S-nitrosylation participates in the regulation of cardiac function by modulating key signaling molecules such as CaMKII, JNK [36], and Hsp90 [37]. For instance, S-nitrosylation of CaMKII plays an important role in β-adrenergic signaling in the heart, where nitrosylation at specific cysteine residues can trigger autonomous activation, thereby affecting calcium release and contributing to arrhythmogenesis [38]. However, both excessive and insufficient levels of S-nitrosylation can lead to pathological states [39,40]. Excessive S-nitrosylation may lead to abnormal activation or inhibition of protein function, whereas insufficient nitrosylation may impair cellular antioxidant capacity and stress response. Thus, the precise regulation of S-nitrosylation is crucial for maintaining cellular homeostasis and preventing disease [41]. Can CSNO ameliorate cardiac dysfunction in the context of renal insufficiency? To address this question, we first added various concentrations of CSNO to cardiomyocytes treated with indoxyl sulfate. The results, which included assessments of cell viability, protein levels, mitochondrial membrane potential, and lipid peroxidation MDA levels, indicated that CSNO supplementation partially mitigated the damage induced by indoxyl sulfate. To further verify the effect of lipid peroxidation on cardiomyocytes, ACAA2 was colocalized with BODIPY, and it was observed that CSNO restored ACAA2 protein levels and reduced the accumulation of lipid intermediates. In addition, we investigated the FOXO4–ACAA2 axis and found that CSNO reduced the RNA level of FOXO4, an upstream regulator of ACAA2, while having no significant effect on the fatty acid transporter CPT2 or the common fatty acid transcription factor PPARA. This suggests that FOXO4 exerts some transcriptional regulation on ACAA2, and CSNO exerts its effect through the FOXO4–ACAA2 axis. In a mouse model, daily aerosol inhalation of CSNO for 20 min resulted in an improvement in cardiac function under renal insufficiency, reducing myocardial hypertrophy and lipotoxic accumulation. Additionally, CSNO significantly attenuated renal fibrosis, although the underlying mechanism remains unclear. It is uncertain whether these effects are due to exogenous NO supplementation or the protective effects of S-nitrosylation of thiol groups. Further exploration of the precise mechanisms is required. Nevertheless, it is evident that aerosolized CSNO can partially restore cardiac function in the setting of renal insufficiency and significantly increase ACAA2 protein levels, thereby improving myocardial lipid metabolism.

## 5. Conclusions

In conclusion, this study provides the evidence that myocardial lipid peroxidation is exacerbated under conditions of renal insufficiency and that restoring ACAA2 protein levels can mitigate lipid peroxidation in cardiomyocytes. Moreover, excessive activation of FOXO4 is detrimental to lipid accumulation in cardiomyocytes, as FOXO4 is involved in the regulation of ACAA2 protein expression. Additionally, we found that CSNO aerosol inhalation effectively improves cardiac function by restoring ACAA2 protein levels and reducing myocardial lipid peroxidation, offering a novel therapeutic strategy for clinical intervention.

## Data Availability

The RNA-seq data supporting the findings of this study are openly available in the Gene Expression Omnibus database (GSE106385).

## Supplementary Materials

The following supporting information can be downloaded at https://www.mdpi.com/article/10.3390/biom15030364/s1; Figure S1: Additional supplementary data; Figure S2: Mice oil red O staining images in heart and kidney; Table S1: PCR primer sequences; Table S2: Top 10 in network string_interactions.tsv ranked by MCC method; File S1: Original blot images.

## Abbreviations

These abbreviations are used in the manuscript.

CON	Control
ACAA2	Acetyl-CoA acyltransferase 2
CSNO	S-nitroso-L-cysteine
FOXO4	Forkhead box O4
IS	Indoxyl sulfate
CKD	Chronic kidney diseases
CVD	Cardiovascular diseases
KFRT	Kidney failure replacement therapy
TMAVA	N,N,N-trimethyl-5-aminovaleric acid
HFpEF	Heart failure with preserved ejection fraction
DCM	Diabetic cardiomyopathy
IRAK2	Interleukin 1 receptor-associated kinase 2
DPP4	Dipeptidyl Peptidase 4
SIRT5	Sirtuin 5
ERR	Estrogen-related receptor
ACC2	Acetyl coenzyme A carboxylase 2
EAT	Epicardial adipose tissue
GEO	Gene Expression Omnibus
CPT2	Carnitine palmitoyltransferase 2
ETFDH	Electron transfer flavoprotein dehydrogenase
ACADM	Acyl-CoA dehydrogenase medium chain
ECH1	Enoyl-CoA hydratase 1
MDA	Malondialdehyde
CAMKII	Calcium/calmodulin-dependent protein kinase II
JNK	c-Jun N-terminal kinase
HSP90	Heat-shock protein 90
PPARA	Peroxisome proliferator-activated receptor alpha
