# Supplementary material for: ACAA2 Protects Against Cardiac Dysfunction and Lipid Peroxidation in Renal Insufficiency with the Treatment of S-Nitroso-L-Cysteine
Source: Biomolecules. 2025 Mar 3;15(3):364. doi: 10.3390/biom15030364 (PMC11940541; doi:10.3390/biom15030364)

Figure2a

CPT2-70kDa

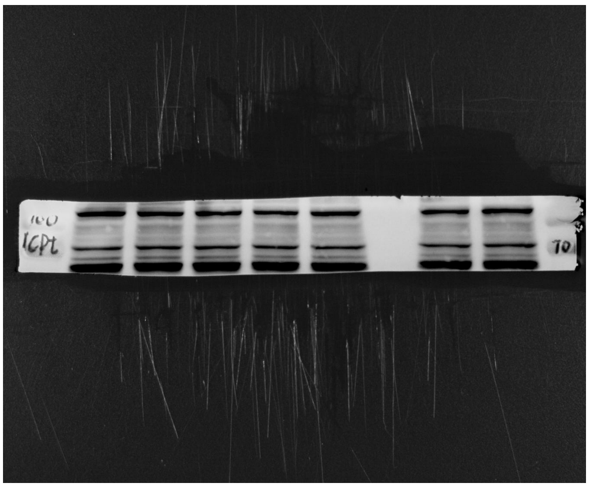

ACADM-45kDa

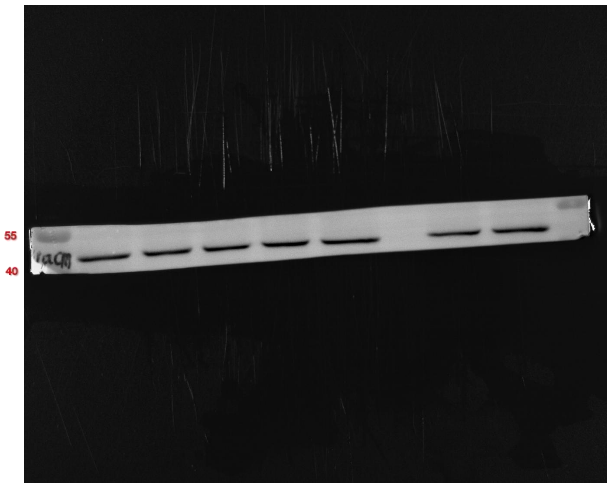

Ech1-36kDa

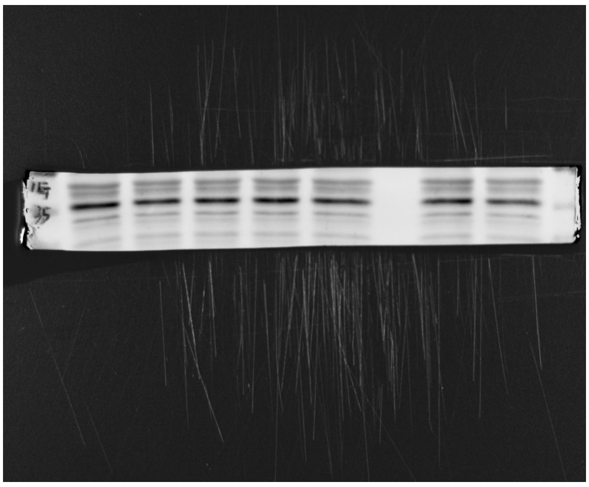

GAPDH-36kDa

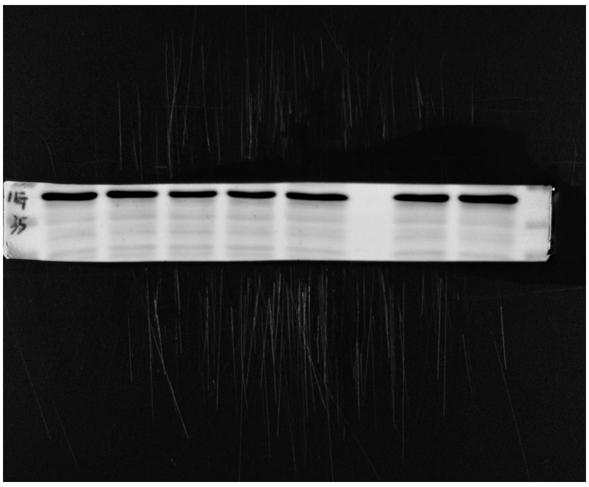

HADHA-76kDa

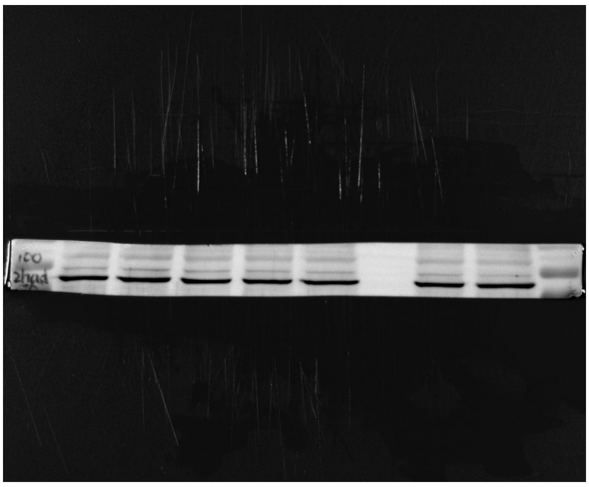

ACAA2-42kDa

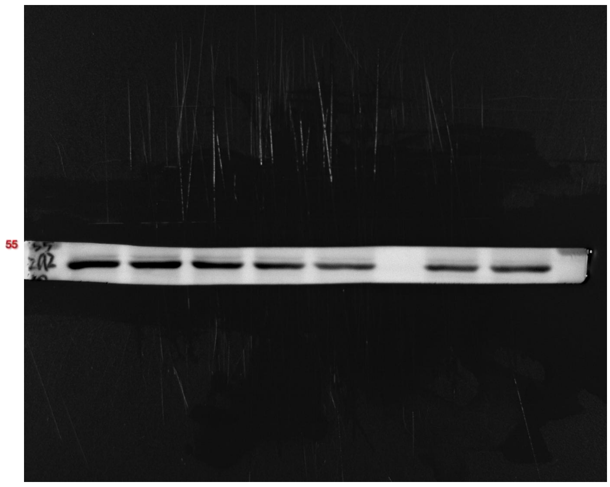

ETFDH-64kDa

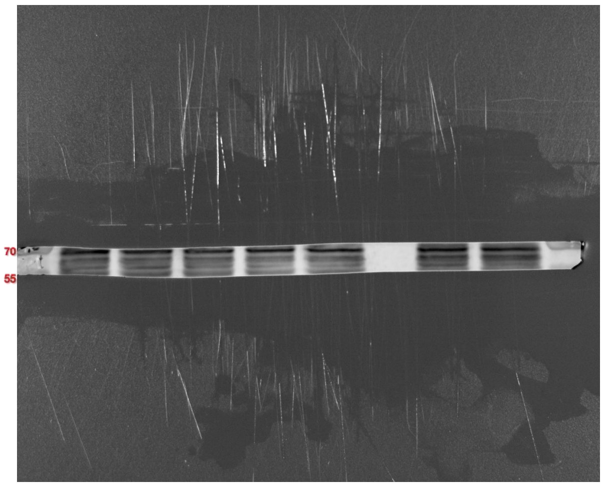

GAPDH-36kDa

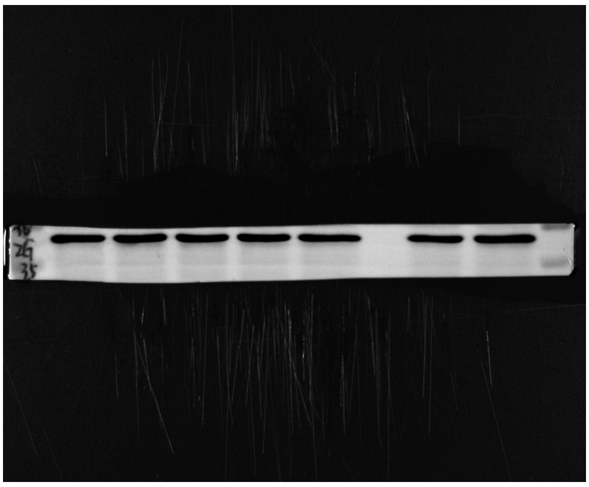

Figure3a

ACAA2-42kDa

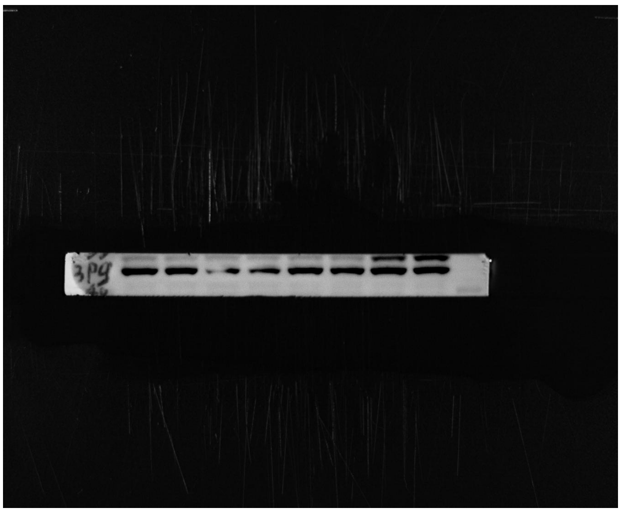

GAPDH-36kDa

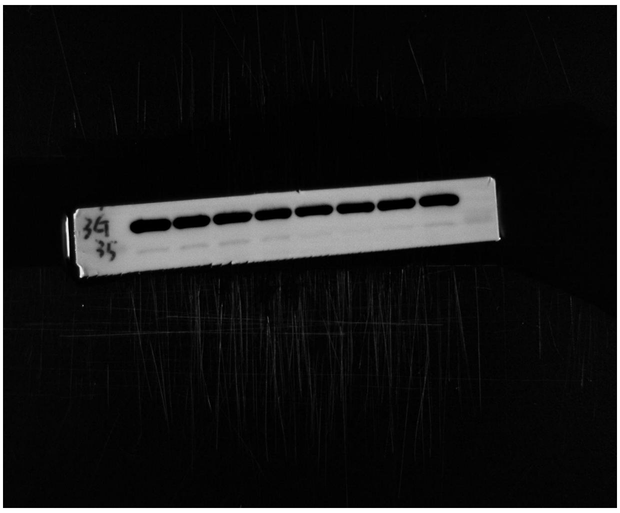

Figure4b

FOXO4-65kDa

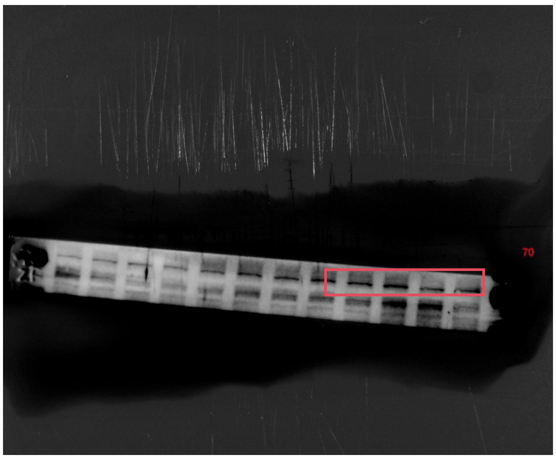

ACAA2-42kDa

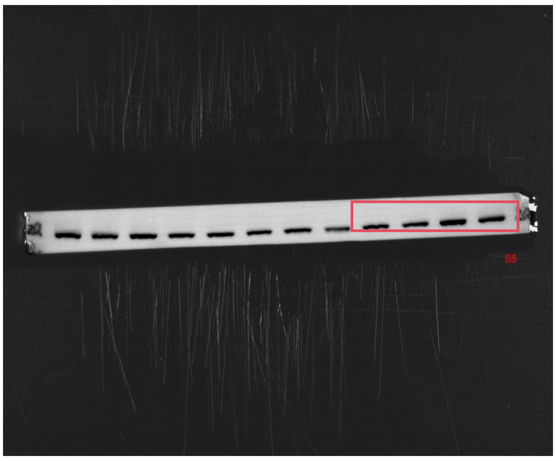

GAPDH-36kDa

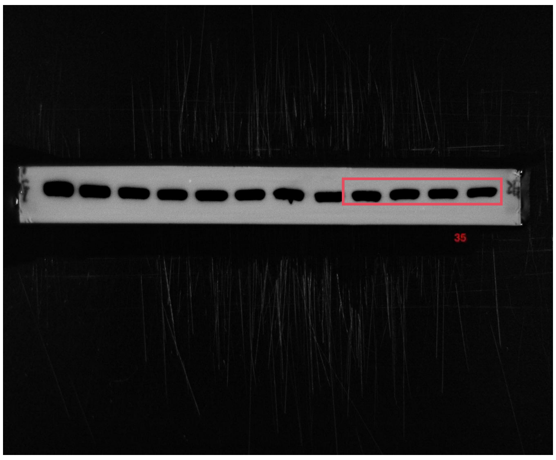

Figure6b

FOXO4-65kDa

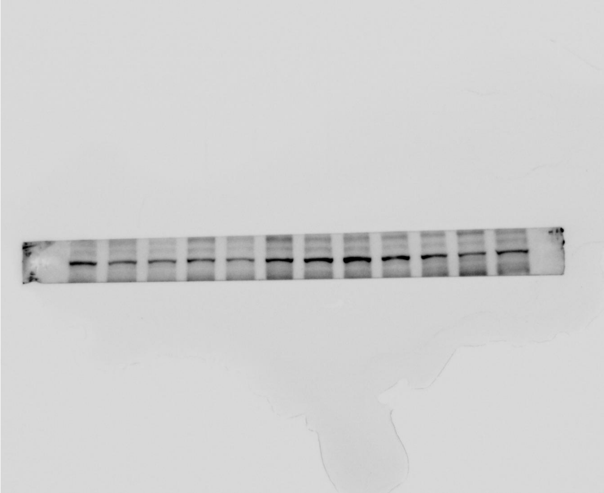

ACAA2-42kDa

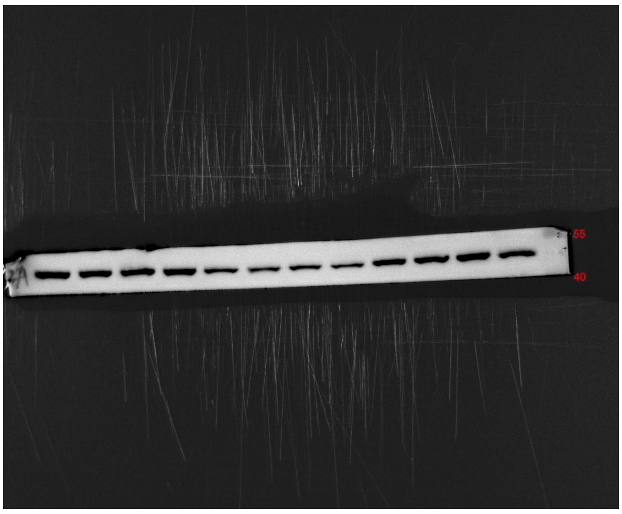

GAPDH-36kDa

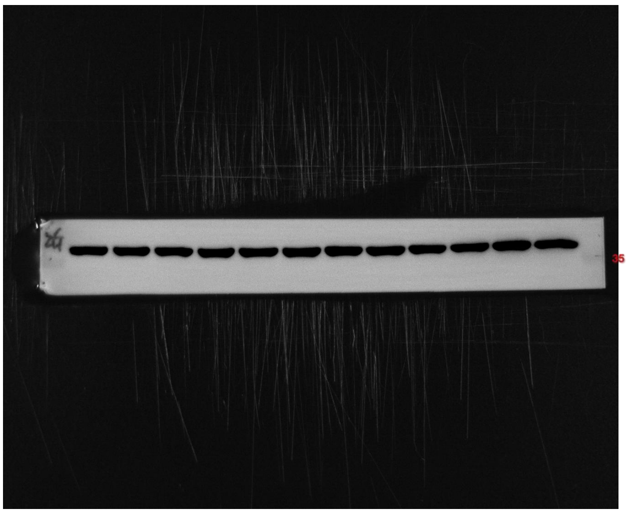

Figure8d

Heart-FOXO4-65kDa

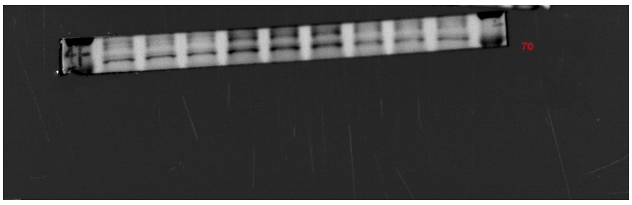

Heart-ACAA2-42kDa

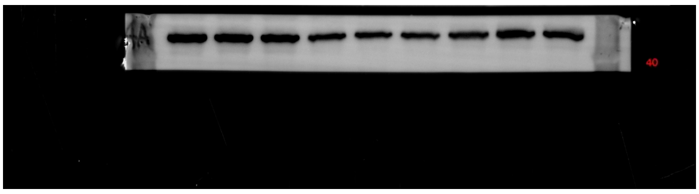

Kideny-Vinculin-124kDa

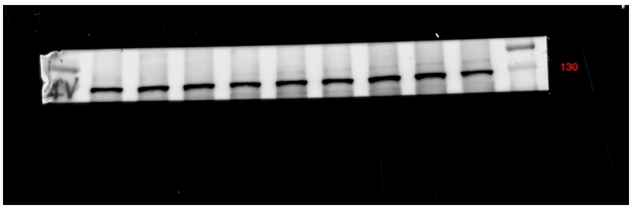

Kidney-FOXO4-65kDa

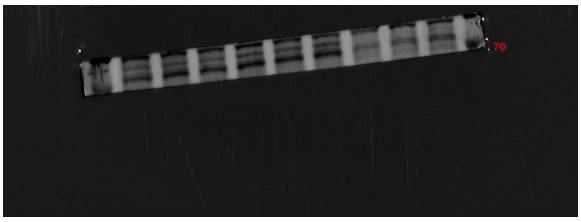

Kidney-ACAA2-42kDa

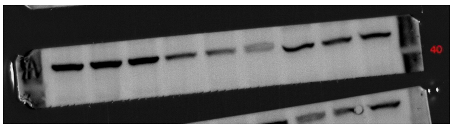

Kideny-Vinculin-124kDa

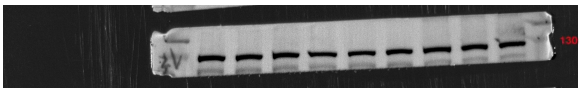

Supplement: Supplementary file 1 [file biomolecules-15-00364-s001.zip › biomolecules-3441400-original images.pdf]
