# Supplementary material for: ACAA2 Protects Against Cardiac Dysfunction and Lipid Peroxidation in Renal Insufficiency with the Treatment of S-Nitroso-L-Cysteine
Source: Biomolecules. 2025 Mar 3;15(3):364. doi: 10.3390/biom15030364 (PMC11940541; doi:10.3390/biom15030364)
Supplement: Supplementary file 1 [file biomolecules-15-00364-s001.zip › Supplementary material.pdf]

Supplementary Material

Supplementary Figure S1

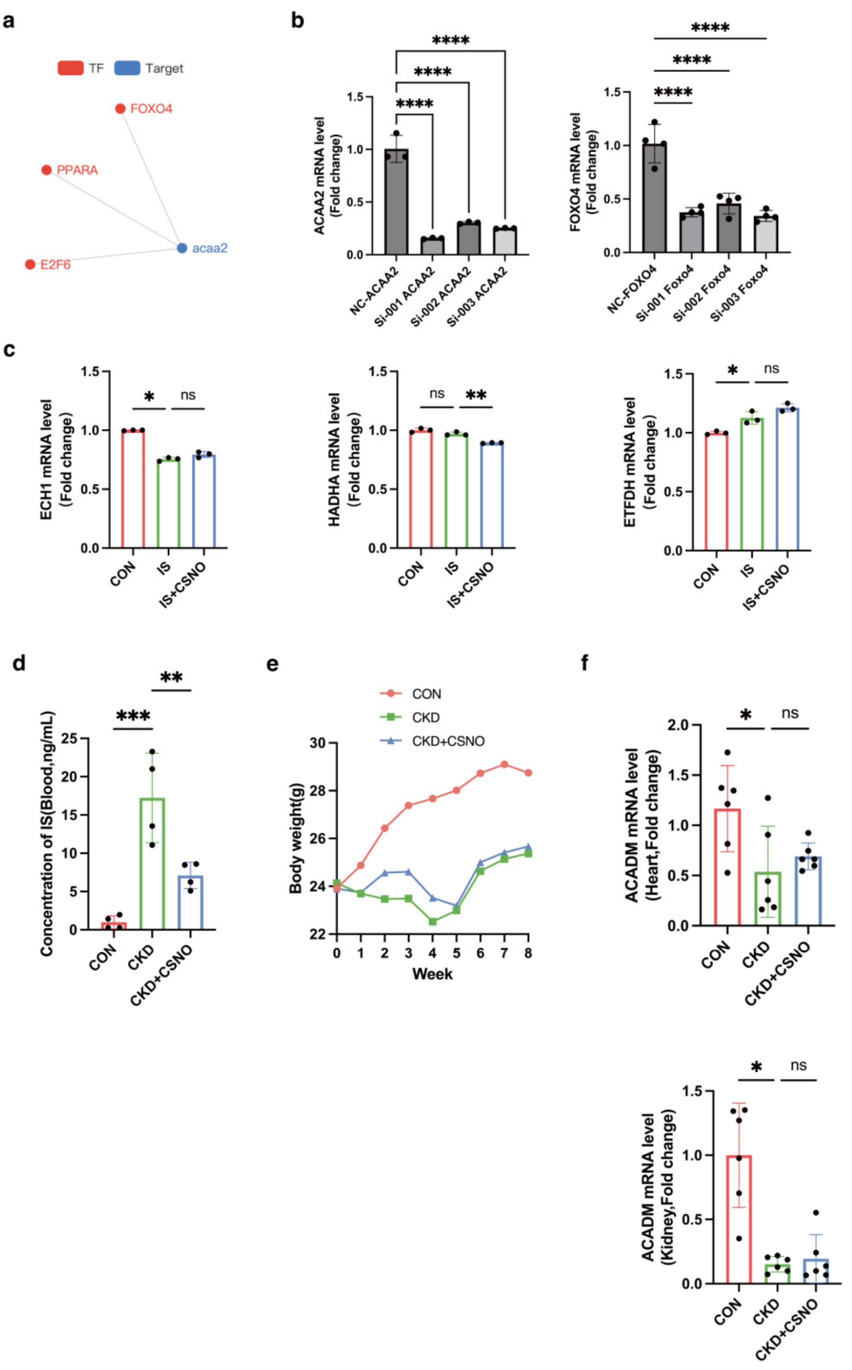

Supplementary Figure S1: (a)Transcription Factor Prediction Map; (b)mRNA levels in AC16 cells with si-ACAA2 and si-FOXO4,n=3-4; (c)mRNA levels in AC16 cells with CSNO treated,n=3; (d) Concentration of indoxyl sulfate in C57BL/6 mice blood samples,n=4; (e)Cronogram of body weight changes in mice,n=10; (f)RNA levels of the ACADM gene in heart and kidney,n=6,*p<0.05, **p<0.01, ***p<0.001, ****p<0.0001, ns: no significant, One-way ANOVA.

Supplementary Figure S2

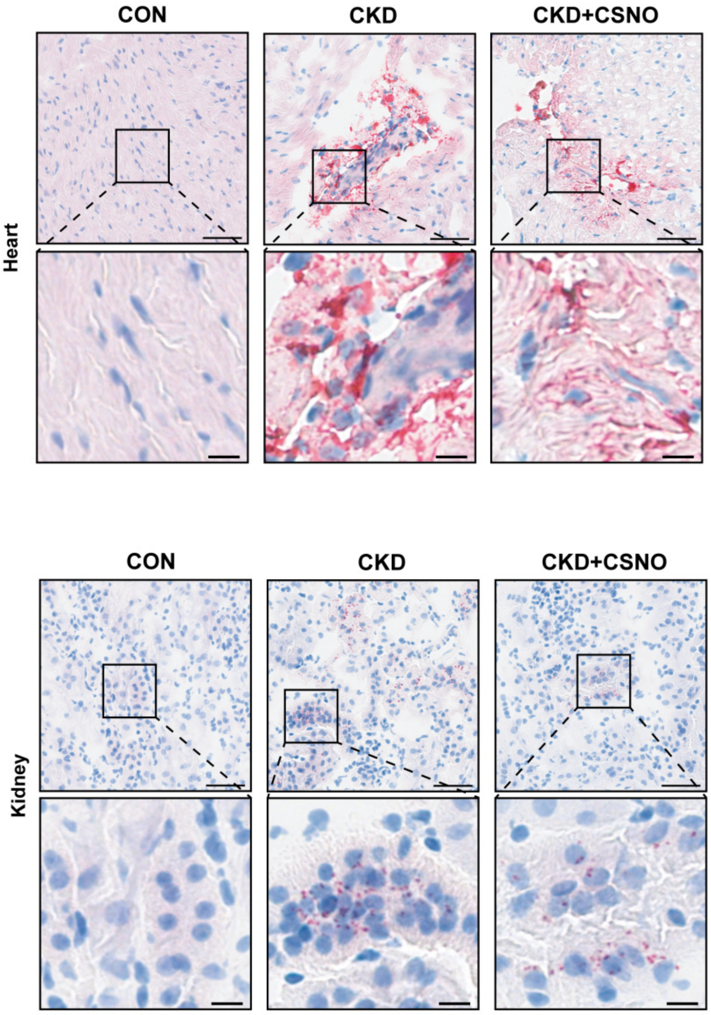

Supplementary Figure S2: Oil Red O staining of mice heart and kidney (long scale bar: 50 µm, short scale bar: 10 µm).

Supplementary Table S1

Table S1 PCR primer sequences

Gene	Primer Type	Sequence(5'->3')
(H)CPT2	Forward	CAGGGCTTTGACCGACACTTG
(H)CPT2	Reverse	GTGCTCGTGGACAGGACATTG
(H)ACADM	Forward	GCCAGCGATGTTCAGATACTAGAG
(H)ACADM	Reverse	CAACAGCACCAGCAGCTACTAC
(H)HADHA	Forward	GGGTGTGCCTGCTGCTTTG
(H)HADHA	Reverse	GGTATTCTATTGTCCGTTCCTCTGG
(H)ACAA2	Forward	ACATAACTTCACACCACTGGCAAG
(H)ACAA2	Reverse	TGATAGCAGGGACAGGACCAATAC
(H)ETFDH	Forward	CTCAAGCCAGCCAAGGATTGC
(H)ETFDH	Reverse	TCAGAGCCACAGATGACAAGAGG
(H)FOXO4	Forward	AACCGTGAAGAAGCCGATATGTG
(H)FOXO4	Reverse	CCTCCGCCAGCACCTCAG
(H)PPARA	Forward	CCTCCTCGGTGACTTATCCTGTG
(H)PPARA	Reverse	GCGTGGACTCCGTAATGATAGC
(M)FOXO4	Forward	CCAGCCATGACAGAATGCCTCAG
(M)FOXO4	Reverse	TGAAGTCCAGTCCCTCACCATCC
(M)ACAA2	Forward	TGCGAAGCGAACACCCTTTGG
(M)ACAA2	Reverse	AGCAGCCCTGGCAGCAAATTC
(M)PPARA	Forward	ACGATGCTGTCCTCCTTGATGAAC
(M)PPARA	Reverse	GATGTCACAGAACGGCTTCCTCAG
(H)GAPDH	Forward	GACAGTCAGCCGCATCTTCT
(H)GAPDH	Reverse	GCGCCCAATACGACCAAATC
(M)GAPDH	Forward	TCTGACGTGCCGCCTGGAG
(M)GAPDH	Reverse	GTGGAAGAGTGGGAGTTGCTGTTG

Supplementary Table S2

Table S2 Top 10 in network string_interactions.tsv ranked by MCC method

Rank	Name	Score
1	Acaa2	2.3574079E7
2	Hadhb	2.3522765E7
3	Hadha	2.3498162E7
4	Acadvl	2.3483815E7
5	Ech1	2.3482886E7
6	Acadm	2.333404E7
7	Etfa	2.3327004E7
8	Etfdh	2.222758E7
9	Eci1	1.541736E7
10	Cpt2	1.4809513E7
